# Maternal E-Cigarette Vaping Drives Persistent Reprogramming of Bone Marrow Hematopoietic and Mesenchymal Stem Cells and Promotes Transcriptional and Metabolic Dysregulation-Associated Inflammaging and Disease Risks in Rat Offspring

**DOI:** 10.3390/cells15171521

**Published:** 2026-08-24

**Authors:** Jeffrey Xiao, Brandon Park, Yong Li, Samiksha Wasnik, Farzad Daniel Fattah, Scott Lee, Kevin Codorniz, Laren Tan, Andrew Chang, Luis Saca, Pamela Lobo Moreno, Michael Matus, Saied Mirshahidi, Raja R. Narayan, Hamid M. Said, Hamid Mirshahidi, Mark E. Reeves, Hisham Abdel-Azim, Huynh Cao, Subburaman Mohan, David J. Baylink, Yi Xu

**Affiliations:** 1 Division of Discovery, Innovation and Regenerative Medicine, Department of Medicine, School of Medicine, Loma Linda University, Loma Linda, CA 92354, USAdanielmd100@gmail.com (F.D.F.);; 2 Lawrence D. Longo MD Center for Perinatal Biology, Department of Basic Sciences, School of Medicine, Loma Linda University, Loma Linda, CA 92354, USA; 3 Division of Endocrinology, Diabetes & Metabolism, Department of Medicine, School of Medicine, Loma Linda University, Loma Linda, CA 92354, USA; 4 Department of Medicine, School of Medicine, Loma Linda University, Loma Linda, CA 92354, USA; 5 Biospecimen Laboratory, Department of Medicine and Basic Sciences, School of Medicine, Loma Linda University, Loma Linda, CA 92354, USA; 6 Division of Hematology and Oncology, Department of Medicine, School of Medicine, Loma Linda University, Loma Linda, CA 92354, USA; 7 Cancer Center, Loma Linda University, Loma Linda, CA 92354, USA; 8 Division of Surgical Oncology, Department of Surgery, Loma Linda University, Loma Linda, CA 92350, USA; 9 Department of Physiology, Biophysics and Medicine, School of Medicine, University of California, Irvine, CA 92697, USA; 10 Musculoskeletal Disease Center, Veterans Affairs Loma Linda Healthcare System, Loma Linda, CA 92357, USA

**Keywords:** hematopoietic stem cells, mesenchymal stem/stromal cells, maternal vaping, nicotine, epigenetics, mitochondrion, KLF4, Notch1, FTO, RUNX2

## Abstract

Adult hematopoietic stem cells (HSCs) and bone marrow (BM) mesenchymal stem/stromal cells (MSCs) are essential for lifelong hematopoiesis, skeletal homeostasis, immune competence, and tissue regeneration. The use of electronic cigarettes (E-cigs) among women of reproductive age continues to rise, raising concerns about potential adverse developmental effects; however, the long-term consequences of maternal E-cig vaping on offspring BM stem cell function and hematopoietic homeostasis remain incompletely understood. Here, using a rat model of maternal E-cig exposure (containing nicotine) during gestation, combined with longitudinal in vivo analyses and complementary ex vivo studies of human cells, we show that prenatal E-cig exposure is associated with persistent alterations in offspring BM stem cell function and lineage commitment. Gestational E-cig exposure was associated with expansion of the CD11b/c^+^ myeloid-enriched compartment, increased CD90^+^ stromal cells, and impaired osteogenic differentiation in rat offspring. Complementary experiments using primary human cells showed that nicotine exposure was associated with reduced T-cell proliferation and impaired cytotoxic activity in a proof-of-principle co-culture assay. Mechanistically, transcriptomic profiling followed by Gene Ontology and pathway enrichment analyses identified alterations in molecular programs associated with KLF4–Notch1 signaling, mitochondrial biogenesis, inflammation, and stem cell regulation in the BM of E-cig-exposed rat offspring. Changes in CCL11, FTO, and RUNX2 were additionally associated with an inflammatory and aging-related molecular phenotype that persisted from early life into adulthood, although these findings do not establish a causal CCL11–FTO–RUNX2 signaling axis or direct cellular senescence. Collectively, our study provides a phenotypic and mechanistic framework for understanding how maternal E-cig exposure may influence long-term offspring hematopoietic, skeletal, and immune health while highlighting the need for further studies to establish causal molecular mechanisms and determine their relevance to maternal E-cig use in humans.

## 1. Introduction

Nicotine-containing tobacco products have been used by humans since the Stone Age in North America [1] and remain a major public health concern. Although conventional tobacco smoking has declined in recent decades, the use of electronic nicotine delivery systems (e-cigarettes or E-cigs), perceived as a safer replacement, has increased substantially, particularly among adolescents and adult populations [2]. In 2023, an estimated 2.13 million U.S. middle and high school students were reported to be using e-cigarettes [3,4]. Epidemiological investigations have shown that regular use of cigarettes and e-cigarettes among women of reproductive age, including during pregnancy, is not uncommon [5,6]. Animal studies have confirmed that in utero exposure to nicotine—a primary component of both cigarettes and e-cigarettes [7]—can jeopardize fetal health by impairing cardiovascular and brain development in offspring [8,9]. Nicotine is also considered a potential gateway drug, lowering the threshold for addiction to other substances such as marijuana and cocaine [10], thereby compounding future healthcare costs and socioeconomic burden. Yet, because e-cigarette devices were introduced relatively recently, the long-term biological consequences of maternal e-cigarette vaping on the development of offspring organs remain largely unknown [11]. Of particular concern, and almost unexamined, is whether such in utero toxin exposure perturbs the continuous renewal, replenishment, and function of tissue stem cells—processes essential to mammalian health across the lifespan—and whether the active agent is nicotine itself or other constituents of the inhaled aerosol.

Bone marrow (BM) is a crucial organ responsible for producing billions of hematopoietic and immune cells daily, sustaining durable physiological function through rapid cell turnover [12]. Within the BM, hematopoietic stem cells (HSCs) give rise to the blood lineages, including erythrocytes that carry oxygen and T cells that perform immunosurveillance [13,14], while mesenchymal stem/stromal cells (MSCs) differentiate into components of the BM microenvironment—osteocytes and vasculature—that support hematopoiesis [15]. Adult lifestyle risk factors have been linked to dysfunction of HSCs [16], leukemia predisposition [17], MSC senescence, and ultimately, hematologic malignancy [18]. However, whether gestational e-cigarette exposure exerts a comparable, lasting effect on offspring BM stem cells and their niche remains largely unexplored [19].

Mechanistically, epigenetic homeostasis is essential for healthy bone marrow function. During aging, dysregulation of DNA methylation, histone modification, and noncoding RNA expression drives HSC and MSC dysfunction [20], impairing regeneration and contributing to bone and blood disorders, including anemia and leukemia [21]. More recently, epitranscriptomic modification of RNA—particularly, N6-methyladenosine (m^6^A), the most abundant internal mRNA modification—has been shown to govern eukaryotic development and to be associated with numerous human diseases [22]. RNA m^6^A modification is dynamically regulated by “writers” such as METTL3 (methyltransferase-like 3), “erasers” such as FTO (fat mass and obesity-associated protein), and “readers” such as YTHDF1-3 [23]. Altered expression of these regulators reshapes the RNA methylome and the physiological programs it controls [24]. In utero exposure to e-cigarette aerosols has been shown to dysregulate FTO-mediated demethylation and to produce offspring hypertension in later adulthood [25], establishing that maternal vaping can leave a durable, disease-relevant epitranscriptomic mark on offspring health. Issues that have not been addressed include whether maternal e-cigarette exposure likewise reprograms the epitranscriptomic landscape of BM stem cells, and whether an FTO/m^6^A axis acting on the master osteogenic transcription factor RUNX2 [26] thereby compromises their osteogenic and hematopoietic output in developing and adult offspring.

Transcription factors that govern stem cell reprogramming may provide a complementary route to the same phenotype. Krüppel-like factor 4 (KLF4) [27] regulates HSC function, stress-induced myelopoiesis, and lymphoid suppression [28], and dysregulated KLF4 signaling has been linked to immune aging and leukemic transformation [29]; it also helps balance osteoblast-mediated bone formation against osteoclast-mediated resorption [30]. Whether maternal e-cigarette exposure alters KLF4-associated transcriptional programs in offspring BM, biasing the marrow toward myelopoiesis at the expense of osteogenesis and toward an immunosenescent, senescence-associated secretory phenotype [31], is unknown. We therefore profiled offspring BM by flow cytometric cell fate mapping, proteomic cytokine analysis, state-of-the-art imaging, genetic engineering and RNA-sequencing approaches to test whether maternal E-cig exposure induces persistent hematopoietic and transcriptomic alterations.

Clinically, dysregulated myelopoiesis has been implicated in cancer progression, where aberrant expansion of immature myeloid cells remodels the BM niche and drives distal immune suppression and tumor-promoting inflammation [32,33]. Early-life perturbations such as in utero e-cigarette exposure may predispose offspring to analogous hematopoietic imbalances later in life through epigenetic and transcriptional priming of bone marrow stem cells, underscoring the potential long-term clinical significance of maternal vaping.

In the present study, we used a rat model of chronic intermittent e-cigarette exposure during pregnancy and performed longitudinal analyses to determine how prenatal exposure affects bone marrow development and the functional properties of HSCs and MSCs in juvenile and adult offspring. We tested the hypothesis that maternal e-cigarette exposure disrupts offspring bone marrow homeostasis by promoting myeloid-enriched hematopoietic changes while impairing MSC osteogenic potential, accompanied by persistent inflammatory and molecular alterations involving FTO, KLF4, and Notch1. To extend observations from the animal model to cells of human origin, we additionally performed complementary ex vivo experiments using human peripheral blood mononuclear cells (PBMCs) and primary T cells to determine whether selected nicotine-associated inflammatory, metabolic, and immune responses identified within our experimental framework could also be observed in human cells. These human-cell studies were designed to provide complementary mechanistic and translational context rather than to clinically validate the effects of maternal e-cigarette vaping in humans. Collectively, our findings indicate that maternal e-cigarette vaping during pregnancy is associated with an inflammatory, immunosenescent state in offspring bone marrow that extends from development into adulthood and may carry lasting consequences for offspring health.

## 2. Materials and Methods

A complete list of reagents, antibodies, kits, primers, and lentiviral plasmids, together with manufacturers and catalog numbers, is provided in Supplementary Tables S1 and S2. Cell-line information is also provided in the Supplementary Materials. Sample sizes and experimental units are specified for the individual experiments and figure legends, and the in vitro assays were generally performed using biological replicates as indicated.

### 2.1. Animal Model of Chronic Intermittent E-Cigarette Device (CIEC) Exposure

All procedures and protocols used in the present study were approved by the Institutional Animal Care and Use Committee of Loma Linda University, in accordance with the National Institutes of Health (US) Guide for the Care and Use of Laboratory Animals. Time-dated pregnant Sprague–Dawley (SD) rats (N = 10, gestational day 4) were purchased from Charles River Laboratories (Portage, MI, USA). The methods for CIEC exposure have been thoroughly described in the previous studies [9,34]. Briefly, SD rats were housed in a temperature-controlled room with a 12 h light/dark cycle and provided with standard rat food and water. The pregnant rats were randomly divided into two groups. Half of the pregnant rats were exposed to scheduled CIEC inhalation during the dark phase of 12 h in a 12/12 h dark/light circadian cycle from day 4 of gestation until near-term (E21). The other half of the pregnant rats were exposed to saline aerosol (saline control) on the same schedule. Animals were randomly assigned to two groups: (1) CIEC exposure (BluPlus E-cigarette, 2.4% nicotine) or (2) saline aerosol control, both administered during the 12 h dark phase from gestational day 4 to near term (E21). BluPlus, a top five bestselling U.S. e-cigarette brand, was selected to reflect real-world exposure [35].

In a previous study using the same exposure protocol from our collaborators, maternal rat plasma nicotine levels were measured and reported at 10–35 ng/mL and cotinine levels at 100–240 ng/mL, concentrations comparable to those observed in human smokers [36]. In the present study of following the same exposure protocol, pregnant rats were exposed from gestational day 4 to near term (E21) during the 12 h dark phase, with saline aerosol used as the control condition. Experiments were conducted in both male and female P7 offspring (N = 10 CIEC offspring and N = 10 control offspring) and adult offspring (>3 months; N = 12 CIEC offspring and N = 12 control offspring). The P7 time point was selected because neonatal bone marrow remains highly proliferative until approximately the third postnatal week and undergoes perinatal lineage, immunological, and transcriptional reprogramming [37,38].

### 2.2. Human Primary Cell and Cell Line Studies

Peripheral blood samples were obtained from healthy adult human donors (N = 6) under protocols approved by the institutional review board, with informed consent obtained from all participants. Mononuclear cells (PBMCs) were isolated using Ficoll–Paque density-gradient centrifugation according to standard protocols. MV4-11 cells (ATCC CRL-9591, a human acute myeloid leukemia (AML) cell line) and PBMCs were cultured in RPMI-1640 medium (Hyclone, Thermo Fisher Scientific, Waltham, MA, USA) supplemented with 10% heat-inactivated fetal bovine serum (FBS, HyClone) and 1% penicillin–streptomycin at 37 °C in a humidified 5% CO_2_ atmosphere. MV4-11 cells were included as an ex vivo human hematologic malignancy model for selected nicotine-exposed T-cell co-culture and related assays.

### 2.3. Isolation and Expansion of Human T Cells from Healthy Donor Peripheral Blood Samples

The detailed procedures of isolating and culturing human T cells ex vivo have been previously reported [39]. Briefly, CD3^+^ T cells from peripheral blood mononuclear cells (PBMNC) were separated using CD3 microbeads and a MiniMACS^TM^ Separator with an MS Column (Miltenyi Biotech, Auburn, CA, USA) according to the manufacturer’s protocol. CD3^+^ T cells were cultured in an RPMI 1640 culture medium containing 10% fetal bovine serum (FBS, HyClone) with penicillin–streptomycin (100 μg/mL), IL-2 (1000 U/mL, PeproTech, Thermo Fisher Scientific, Waltham, MA, USA), IL-7 (25 ng/mL, PeproTech), IL-15 (25 ng/mL, PeproTech) and Dynabeads^TM^ HumanT-Activator CD3/CD28 (Gibco^TM^, Thermo Fisher Scientific, Waltham, MA, USA) without feeder cells. T cells were grown at 37 °C in a humidified atmosphere containing 5% CO_2_.

### 2.4. Tissue and Cell Harvesting

On postnatal day 7 (P7) or in adulthood, the offspring rats were euthanized by CO_2_ inhalation followed by cervical dislocation. Spleens were excised, measured for length (cm), photographed, and processed immediately. Femurs and tibias were aseptically dissected, and bone marrow was flushed with ice-cold PBS supplemented with 2% FBS using a 25-gauge needle.

### 2.5. Preparation of Single-Cell Suspensions

Spleens were mechanically disrupted through 70 μm cell strainers in RPMI-1640 culture medium. Red blood cells (RBCs) were lysed using ACK lysis buffer for 3 min, room temperature. BM cells were filtered through 40 μm strainers, washed twice with PBS/2% FBS, and counted using trypan blue exclusion. Single-cell suspensions were maintained on ice until staining or continuous in vitro cell culture.

### 2.6. MSC Isolation, Culture, and Differentiation

Adult bone marrow-derived mesenchymal stem/stromal cells (MSCs) from CIEC-exposed (EV) and control offspring were isolated by plastic adherence as previously described [40]. Cells were plated in DMEM with 10% FBS and cultured for 7–10 days with medium changes every 3 days. Non-adherent cells were removed, and adherent MSCs were expanded to passages 3–5. MSCs were characterized by morphology (round-shaped vs. angular-shaped) and CD11b/c expression (CD11b/c^+^ vs. CD11b/c^−^ subsets). CD11b/c^+^ vs. CD11b/c^−^ MSC subsets were isolated by microbead separation as previously described [39]. Briefly, single-cell suspensions were first incubated with anti-CD11b/c-APC for 30 min at 4 °C and washed. The CD11b/c-APC-stained cells were then incubated with anti-APC microbeads (10 μL per 10^7^ cells, 30 min, 4 °C), washed again, and applied to MACS columns placed in a magnetic field. The CD11b/c^−^ fraction was collected in the flow-through, and the retained CD11b/c^+^ cells were subsequently eluted by removing the column from the magnet and flushing with buffer. Both fractions were washed, counted, and assessed for purity before downstream applications.

For osteogenic differentiation, MSCs were cultured in osteogenic medium (DMEM supplemented with 10% FBS, 10 mM β-glycerophosphate, 50 μg/mL ascorbic acid, and 100 nM dexamethasone) for 21 days with medium changes twice weekly. Mineralization was assessed by Alizarin Red S staining (2%, pH 4.2, 20 min). Calcified nodules were photographed and quantified by extracting the dye with 10% cetylpyridinium chloride and measuring absorbance at 562 nm.

### 2.7. Lentiviral Transduction and Overexpression of CCL11 and FTO Transgenes

CCL11 lentivirus (lentiviral expression vector, Cat# EX-Z7461-Lv225, GeneCopoeia, Rockville, MD, USA) was produced in HEK293T cells using standard packaging protocols as previously described [41]. FTO lentiviral particles were purchased from GeneCopoiea (Cat# LPP-Rn18290-Lv105-A00). Primary MSCs or human peripheral blood (hPB) cells or human T cells were transduced at an MOI of 10 in the presence of 8 μg/mL polybrene for 24 h. Transduced cells were selected with puromycin (2 μg/mL) for 48–72 h and expanded. CCL11 or FTO overexpression was confirmed by qPCR and flow cytometry for RUNX2 (Runt-related transcription factor 2) and RANK (Receptor activator of nuclear factor κ B) expression changes.

### 2.8. E-Cigarette Exposure Conditions and Nicotine Treatment

Human PBMCs were cultured at a concentration of 1 × 10^7^ cells/mL and exposed to freshly generated e-cigarette vapor using a CIEC vapor delivery system for 12 h in vitro, followed by cell culture medium replacement and an additional 48 h incubation. Control cells were exposed to filtered air under otherwise identical conditions.

Human PBMCs and T cells were treated with different doses of nicotine, including 10 μM. Treatment of <~1 mM nicotine was reported to have nontoxic effects on human osteoblast cells ex vivo [42,43]. Controls received vehicle only. Treatments were conducted for 48 h before cells and supernatants were harvested for subsequent analysis.

### 2.9. RNA Sequencing (RNA-Seq) and Bioinformatic Analysis

Total RNA was isolated from bone marrow (BM) tissues collected from 4-week-old male offspring rats prenatally exposed to either room air (WT control) or e-cigarette vapor (EV) during gestation. RNA quality and integrity were assessed prior to library preparation. Bulk RNA sequencing was performed by BGI Genomics Americas using three biological replicates per group (N = 3 WT and N = 3 EV offspring). Sequencing libraries were generated according to the manufacturer’s standard protocols and sequenced on an Illumina platform to obtain paired-end reads.

Briefly, total RNA was subjected to high-throughput RNA sequencing using a 150-bp paired-end sequencing strategy, generating approximately 20 million reads per sample. Raw sequencing data were quality-filtered prior to alignment to the rat reference genome used in the original BGI analysis pipeline. Read alignment, gene expression quantification, and downstream bioinformatics analyses were performed using the Dr. Tom Bioinformatics Platform by BGI Americas (Cambridge, MA, USA). Differential gene expression analysis was conducted using the DESeq2 package, and genes with a false discovery rate (FDR)-adjusted *p*-value < 0.05 and an absolute log_2_ fold change ≥ 1 were considered significantly differentially expressed. Differentially expressed genes were subsequently used for downstream pathway and biological interpretation. Gene expression levels were quantified as transcripts per million (TPM), or fragments per kilobase of transcript per million mapped reads (FPKM). Differential gene expression analysis was performed between WT and EV offspring BM samples using standard statistical pipelines. Genes exhibiting significant expression changes were further analyzed using Gene Ontology (GO) and pathway enrichment analyses to identify biological processes and signaling pathways altered by maternal EV exposure. Heatmaps, transcript distribution plots, and pathway enrichment visualizations were generated to characterize global transcriptomic differences between experimental groups. Human T cells were genetically engineered to overexpress FTO (FTO-OE) and compared with control T cells. Total RNA was extracted and subjected to bulk RNA sequencing following the same protocols above (aligned to the human reference genome, GRCh38/hg38) to identify differentially expressed genes between control and FTO-OE T cells. Gene expression profiles were visualized using heatmap analysis, and significantly altered genes were subjected to Kyoto Encyclopedia of Genes and Genomes (KEGG) pathway enrichment analysis to identify biological pathways regulated by FTO overexpression.

### 2.10. Quantitative PCR (qPCR)

Treated cells were collected for RNA isolation and qPCR analysis as previously described [44]. Total RNA was isolated using the RNeasy Mini Kit (Qiagen, Germantown, MD, USA) according to the manufacturer’s instructions. First-strand cDNA was synthesized using the SuperScript III Reverse Transcriptase (Invitrogen, Waltham, MA, USA). qPCR was performed using an Applied Biosystems 7900HT Real-Time PCR machine (Waltham, MA, USA) with primers specific for CDK1, Cyclin B1 (CCNB1), and CCL11 and appropriate housekeeping genes. Sequences of the primers used in this study are available in Supplementary Table S2. The PCR conditions were 10 min at 95 °C followed by 40 cycles of 10 s at 95 °C and 15 s at 60 °C. The relative expression level of each gene was determined using the ΔΔCt method and normalized to β-actin.

### 2.11. Flow Cytometry and Immunophenotyping

Single-cell suspensions of experimental groups were examined for the expression of cell surface biomarkers and intracellular proteins using multichromatic flow cytometry (FC), with a detailed gating strategy as previously described [45,46]. About 0.5~1 × 10^6^ cells were resuspended in 100 µL FC buffer (PBS containing 1% FBS and 0.05% sodium azide) and stained with various, fluorescently conjugated antibodies specific for the desired cell surface proteins at 4 °C for 30 min. Isotype controls and fluorescence minus one (FMO) controls were performed to exclude non-specific binding and autofluorescence. Antibody panels included CD45 (hematopoietic cells), CD90 (stromal cells), CD11b/c (myeloid cells), CD3 (T cells), CD34 (hematopoietic stem/progenitor cells), RUNX2 (osteogenic marker), RANK (osteoclastogenic marker), and FTO (m^6^A demethylase). The surface-stained cells were then fixed and permeabilized using the appropriate reagents (BD Pharmingen Cytofix/Cytoperm buffer) and further stained with appropriate fluorescent-conjugated antibodies specific to the desired intracellular proteins, such as RUNX2, at 4 °C for 2 h in permeabilizing buffer (BD Perm/Wash buffer). Concentrations of the antibodies were used per the manufacturers’ recommendations (Supplementary Table S1). Finally, the stained cells were washed twice in permeabilizing buffer and twice in FC buffer before analysis on the BD FACSAria II. Data were analyzed using FlowJo software (v10.6, Tree Star Inc., Ashland, OR, USA).

### 2.12. Western Blotting (WB) Analysis

BM cells were collected for WB as previously described [47]. Briefly, cells were homogenized in an ice-cold cell lysis buffer composed of 20 mM HEPES (pH 7.5), 10 mM KCl, 1.5 mM MgCl_2_, 1 mM ethylenediaminetetraacetic acid, 1 mM dithiothreitol, 1 mM phenylmethylsulfonyl fluoride, 2 µg/mL of aprotinin, and 10 µg/mL of leupeptin, followed by incubation on ice for 30 min. The homogenates were ultrasonicated and centrifuged at 20,000× *g* for 30 min at 4 °C. Samples with equal quantities of protein were loaded onto a 10% SDS–polyacrylamide gel and separated by electrophoresis at 100 V for 1 h. Protein bands on the gel were then transferred onto Immobilon-P membranes (Millipore Corporation, Burlington, MA, USA) and were blocked with 5% non-fat milk (1 h, room temperature), incubated with primary antibodies against FTO (m^6^A demethylase) overnight at 4 °C, then with Amersham HRP-conjugated secondary antibodies (1 h, room temperature) (Cytiva, Marlborough, MA, USA). Bands were visualized using an ECL substrate and quantified by densitometry using ImageJ 1.54p, with normalization to GAPDH loading controls.

### 2.13. Cytokine and Proteomic Profiling

To examine the cause of increased BM myelopoiesis, we collected blood plasma samples from adult CIEC-exposed offspring and control rats for rat cytokine profile assays. This cytokine assay (Rat XL Cytokine Array Kit, catalog # ARY030, R&D, Minneapolis, MN, USA) included 79 different antibodies against essential cytokines, chemokines, and growth factors, with their corresponding coordinates listed on the manufacturer’s datasheet. Supernatants from nicotine-treated human peripheral samples were analyzed using human cytokine proteomics arrays (Human XL Cytokine Array Kit, catalog # ARY022B, R&D, Minneapolis, MN, USA) including 105 different antibodies against essential cytokines, chemokines, and growth factors, with their corresponding coordinates listed on the manufacturer’s datasheet. CCL11 (Eotaxin), MIF (macrophage migration inhibitory factor), and DPPIV (CD26) levels were quantified with ImageJ software along with all the other listed cytokines from the manufacturer’s datasheet. Proteomic data were normalized to total protein and analyzed using appropriate statistical software.

### 2.14. Seahorse Assay

Different experimental groups (48 h nicotine treatment) were analyzed using a Seahorse XFe24 Analyzer (Agilent, Santa Clara, CA, USA). The experimental procedure and drug concentrations were applied by following our previous report [48] and the manufacturer’s protocols.

### 2.15. Cell Morphology Analysis

MV411 cells were seeded at 5 × 10^5^ cells/mL in 6-well tissue culture plates and exposed to freshly generated e-cigarette vapor using the custom vapor delivery system described above or exposed to filtered air (control) for 12 h, followed by 48 h of recovery in fresh culture medium. Cell morphology was assessed using phase-contrast light microscopy. The number of cells exhibiting elongated morphology was counted and expressed as the number of elongated cells per 1000 total cells evaluated. To ensure statistical robustness, a minimum of 1000 cells per treatment condition per replicate were analyzed and representative images from each treatment group were captured and preserved for illustrative purposes.

### 2.16. Micro-Computed Tomography (μCT)

Femoral bones from experimental groups were harvested and fixed in 4% paraformaldehyde. Samples were scanned using a μCT system (VivaCT 40, Scanco Medical, Southeastern, PA, USA) at a voxel resolution of 10.5 μm, as previously described [49]. Briefly, the region of interest was manually contoured along the bone perimeter. Images were reconstructed and analyzed using the manufacturer-provided 2D and 3D analysis software (Scanco Medical, Southeastern, PA, USA). Quantitative morphometric parameters, including bone volume per total volume (BV/TV), connectivity density (Conn.D), and bone mineral density (BMD), were calculated. Percent fat content was assessed separately using a Faxitron radiography system (Hologic, Marlborough, MA, USA).

### 2.17. Statistical Analysis

Data are presented as mean ± standard deviation unless otherwise indicated. Statistical significance was determined using a two-tailed Student’s *t*-test for two-group comparisons, whereas one-way or two-way ANOVA followed by Tukey’s post hoc test was used for multiple-group comparisons. *p*-values < 0.05 were considered statistically significant. The experimental unit and biological replicate number are reported in the corresponding figure legends.

## 3. Results

### 3.1. Maternal Vaping During Pregnancy Promotes Persistent Myelopoiesis in the Bone Marrow In Vivo

**Figure 1:**
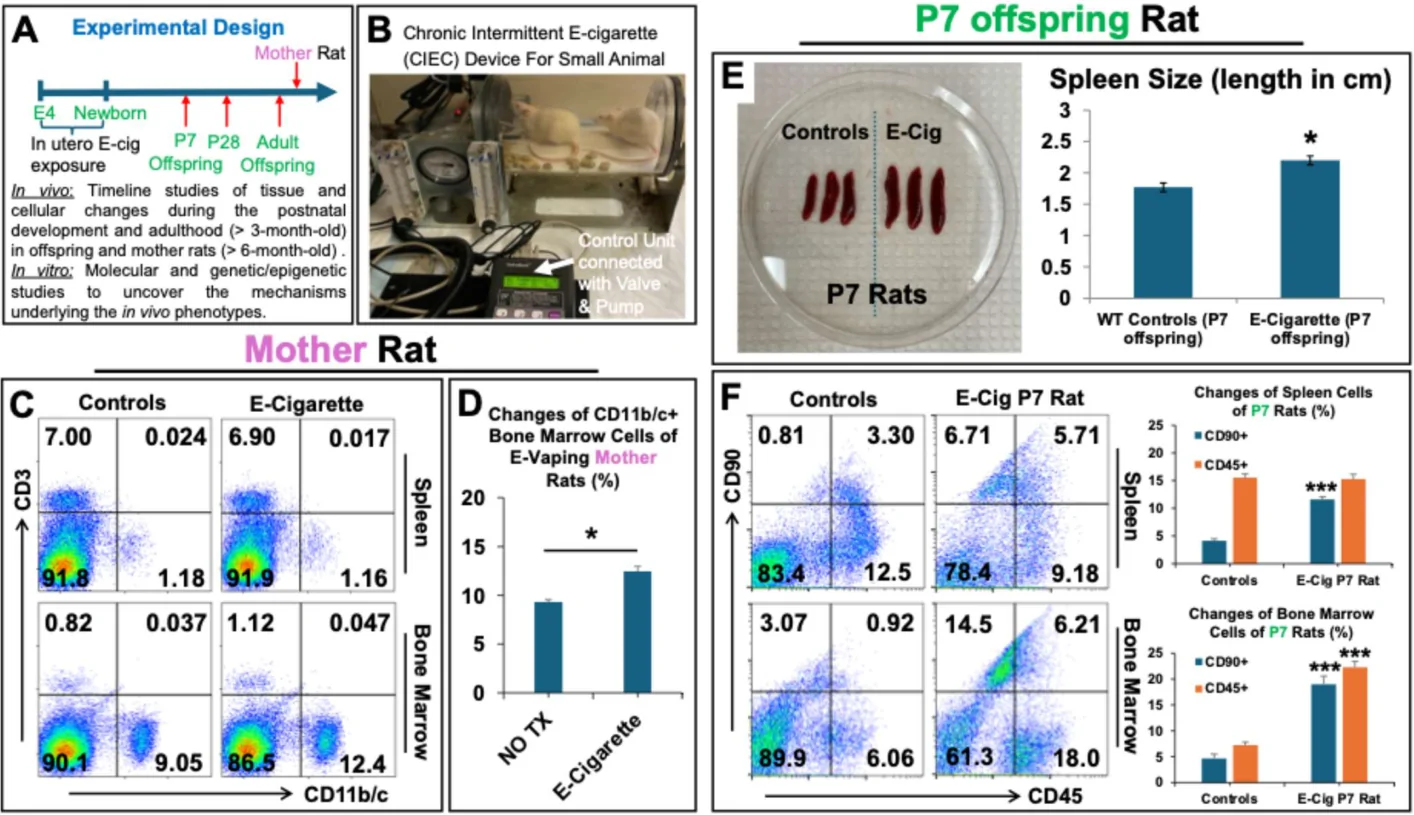
Maternal e-cigarette vaping during pregnancy promotes persistent myelopoiesis in the bone marrow (BM) of mother rats and induces organ hypertrophy and cellular alterations in both the spleen and BM of P7 offspring in vivo. (**A**) Experimental design and timeline showing in utero E-cig exposure from embryonic day 4 (E4) of rat development to newborn rat at postnatal day 0 (P0). Postnatal day 7 (P7) and adult offspring along with mother rats were sacrificed for both in vivo and in vitro analyses. (**B**) A CIEC device where aerosol exposure is controlled by a programmable control unit (white arrow). Pregnant animals are in a chamber connected to a smoke outlet. (**C**) Flow cytometry analyses show E-cig exposure increased the percentage of CD11b/c^+^ myeloid cells in the mother rat’s bone marrow (BM). (**D**) Representative graph of E-vaping mother rats displaying significantly increased CD11b/c^+^ myeloid cells in the BM compared to control mother rats. (**E**) Image of visibly larger spleens in the E-cig P7 offspring (**left**) and representative graph of significantly increased spleen length in cm of the E-cig P7 offspring rat group compared to the wild-type (WT) control P7 offspring rat group (**right**). (**F**) Flow cytometry analyses (**left**) and representative graph (**right**) show significantly increased CD90^+^ stromal cells in the spleen of the E-cig P7 offspring rat group and significantly increased CD90^+^ stromal cells and CD45^+^ hematopoietic immune cells in the BM of the E-cig P7 offspring rat group compared to control offspring rats. Where applicable, data are presented as means ± SEM. * *p* < 0.05, *** *p* < 0.005, N = 3 biological replicates.

To investigate the long-term biological effects of maternal e-cigarette (E-cig, nicotine-containing) vaping during pregnancy on offspring organs including bone marrow (BM) and spleen, we applied the previously reported chronic intermittent e-cigarette exposure (CIEC) rat model designed to approximate nicotine levels achieved by human e-cig users [9,34]. Briefly, pregnant female SD rats were exposed to E-cigarette aerosol from embryonic day 4 (E4) to birth, via a pressurized air activating system, and continuously monitored (Figure 1A,B). Some P7 offspring rats were sacrificed, while the mother rats and the remaining offspring rats that became adults were sacrificed at least 3 months post-CIEC exposure in utero for pathophysiological analyses (experimental design, Figure 1A). Flow cytometry (FC) analysis showed that e-cigarette-exposed mothers (six months post-delivery) had a significantly increased CD11b/c^+^ cell population (a biomarker for myeloid cells and macrophages [50]) in the BM compared to control mother rats (9.09% vs. 12.45%, Figure 1C,D). These findings suggest that gestational nicotine exposure induced persistent myelopoiesis in the maternal bone marrow long after delivery.

### 3.2. Maternal Vaping During Pregnancy Induces Spleen Hypertrophy and Cellular Alterations in P7 Offspring Spleen and Bone Marrow In Vivo

Next, we investigated the effect of gestational nicotine exposure on the development of offspring. The P7 E-cig-exposed offspring rats exhibited significantly enlarged spleens with an average length of 2.2 cm compared to ~1.7 cm in the control group (Figure 1E). Consistent with the splenic hypertrophy, FC analysis revealed increased CD90^+^ stromal cells in the spleen of P7 offspring versus the control group (12.42% vs. 4.11%). Furthermore, FC analysis revealed marked increases in both CD90^+^ stromal cells (20.1% vs. 3.99%) and CD45^+^ hematopoietic cells (24.21% vs. 6.98%) in the bone marrow of P7 offspring versus control group, respectively (Figure 1F). These findings indicate that maternal E-cig vaping can affect both gross organ size and cellular composition in the developing spleen and BM of offspring.

### 3.3. Maternal Vaping During Pregnancy Promotes CD11b/c^+^ Myelopoiesis and CD90^+^ Mesenchymal Stromal Expansion in the Bone Marrow of Adult Offspring

**Figure 2:**
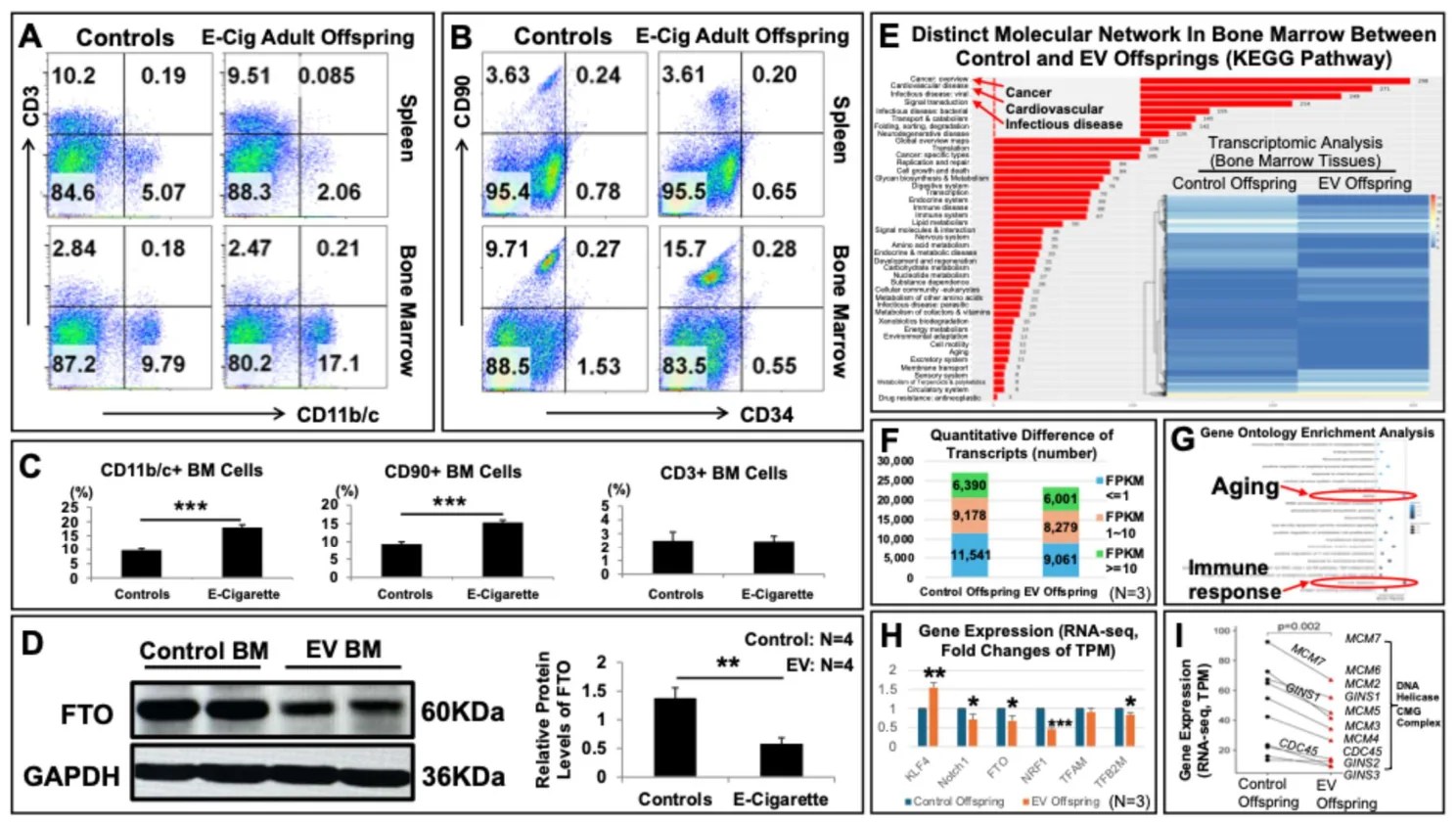
Prenatal nicotine exposure promotes myeloid-enriched populations and transcriptomic alterations in vivo in rat offspring bone marrow. (**A**) Flow cytometry reveals increased CD11b/c^+^ myeloid-enriched cells in the BM, with a comparable CD3^+^ T-cell population in the BM and spleen of adult offspring exposed to E-cig aerosol in utero. (**B**) Flow cytometry reveals significantly increased CD90^+^ stromal cells in the BM of adult offsprings exposed to E-cig aerosol in utero, with a trend towards reduced CD34^+^ hematopoietic stem and progenitor cells in the BM and spleen. (**C**) Representative graphs show a significant increase in CD11b/c^+^ myeloid-enriched and CD90^+^ stromal BM cells but similar CD3^+^ T-cell populations in the E-cig-exposed adult offspring rat group compared to controls. (**D**) Western blotting analysis of bone marrow tissues reveals a significant reduction in protein expression of FTO, an RNA demethylase, in the E-cig-exposed adult offspring group compared to controls. Densitometric quantification confirms altered protein expression consistent with the transcriptomic findings. (**E**) RNA-sequencing analysis identifies distinct transcriptomic signatures and molecular networks in bone marrow cells of E-cig offspring compared with control offspring. (**F**) Transcriptomic profiling reveals reduced overall transcript abundance in BM cells of E-cigarette offspring relative to controls. (**G**) Gene Ontology enrichment analysis demonstrates pathways associated with aging and immune regulation (red circles) in bone marrow cells of E-cig offspring. (**H**) RNA-sequencing analysis reveals increased KLF4 and decreased Notch1 and FTO expression, together with altered expression of mitochondrial biogenesis regulators NRF1 and TFB2M, in BM cells of E-cigarette offspring compared with controls. (**I**) Transcriptomic analysis reveals reduced expression of DNA helicase CMG-complex components in BM cells of E-cigarette offspring compared with controls. Where applicable, data are presented as means ± SEM. * *p* < 0.05, ** *p* < 0.01, *** *p* < 0.005, N = 3 biological replicates.

To study longer-term biological consequences of maternal e-cigarette vaping during pregnancy, we then investigated adult offspring (>3 months old). There was no significant difference in spleen sizes between E-cig-exposed adult offspring and control groups. Consistent with enhanced hematopoietic and mesenchymal expansion in the bone marrow (BM) of the developing P7 offspring rats, FC analysis also revealed significantly increased CD11b/c^+^ myeloid cells (17.31% vs. 9.97%) and increased CD90^+^ stromal cells (15.98% vs. 9.98%) in the BM of E-cig adult offspring compared to the control group (Figure 2A–C). In contrast, there were slight but non-significant decreased changes in CD3^+^ T cells in the spleen (9.6% vs. 10.39%) and BM (2.68% vs. 3.02%) in E-cig adult offspring compared to the control group (Figure 2A). Also, FC analysis revealed slightly reduced but non-significant changes in CD34^+^ hematopoietic stem and progenitor cells in the spleen and BM (0.85% vs. 1.02% and 0.83% vs. 1.8%, respectively) in E-cig exposed adult offspring compared to the control group (Figure 2B), which is consistent with a previous report of impaired hematopoiesis in a mouse E-cig model [17].

Cigarette smoke has been reported to impair N6-methyladenosine (m^6^A) RNA methylation in macrophages by downregulating FTO (fat mass and obesity-associated protein, a demethylase regulator of m^6^A), leading to chronic airway inflammation [51]. To investigate if these mechanisms underlie E-cig-altered BM phenotypes, we assessed FTO protein expression and observed significantly reduced FTO expression in the BM of E-cig-exposed rat offspring (Figure 2D). This finding is consistent with reports of diminished FTO expression promoting human monocyte activation [52]. Together, these results indicate that maternal E-cig vaping during pregnancy alters hematopoietic and stromal stem cell composition in adult offspring BM, potentially via FTO-related epigenetic dysregulation.

### 3.4. Transcriptomic Analysis of Offspring Bone Marrow Cells Identified Mechanistic Evidence Linking Gestational Nicotine Exposure to Transcriptional and Metabolic Stress-Mediated Hematopoietic Alterations

To validate our Western blot and qPCR findings at the transcriptomic level, we performed bulk RNA sequencing on bone marrow cells isolated from E-cigarette-exposed offspring and age-matched control offspring. Global transcriptomic analysis revealed distinct molecular signatures and gene expression patterns between the two groups, indicating that prenatal nicotine exposure was associated with persistent transcriptional reprogramming in the offspring bone marrow compartment (Figure 2E). Notably, E-cigarette offspring exhibited a marked reduction in overall transcriptional abundance compared with controls (Figure 2F). Gene Ontology enrichment analysis further identified multiple differentially regulated pathways associated with aging, immune responses, and cellular homeostasis (Figure 2G). Consistent with our protein and qPCR analyses, integrated RNA-sequencing data demonstrated significantly reduced FTO and dysregulated gene expression of KLT4 and Notch1 in bone marrow cells from E-cigarette offspring (Figure 2H). These findings support the notion that gestational nicotine exposure promotes inflammation-associated and aging-associated molecular alterations in bone marrow cells of rat offspring but do not by themselves demonstrate cellular senescence or premature hematopoietic aging.

The mitochondrion is a central hub for many fundamental physiological processes from ATP energy production to cell growth and maintenance, and it plays essential roles in hematopoietic aging [53] and a myriad of human diseases [41]. The transcriptomic analysis revealed significantly reduced expressions of key drivers of mitochondrial biogenesis, including nuclear respiratory factor 1 (NRF1) and mitochondrial transcription factor B2 (TFB2M) in the BM of E-cig-exposed rat offspring in vivo (Figure 2H).

DNA helicase CMG complex—the CDC45-MCM2-7-GINS assembly, is a conserved core architecture of the eukaryotic replisome, responsible for unwinding the DNA double helix and driving DNA replication fork progression and genomic duplication [54]. Growing evidence suggests that the reduction of CMG helicase components contributes significantly to dysfunction and aging of hematopoietic stem cells [55]. The transcriptomic analysis revealed significantly reduced gene expressions of most CMG helicase family members (Figure 2I).

Taken together, these findings provide preliminary mechanistic evidence linking in utero nicotine exposure-impaired epigenetic and pluripotency regulators, dysregulation of mitochondrial biogenesis, and a scarcity of CMG helicase components to altered phenotypes of BM stem cells observed in the E-cigarette offspring.

### 3.5. Altered Mesenchymal Stem/Stromal Cell (MSCs) Populations in Adult Offspring BM

**Figure 3:**
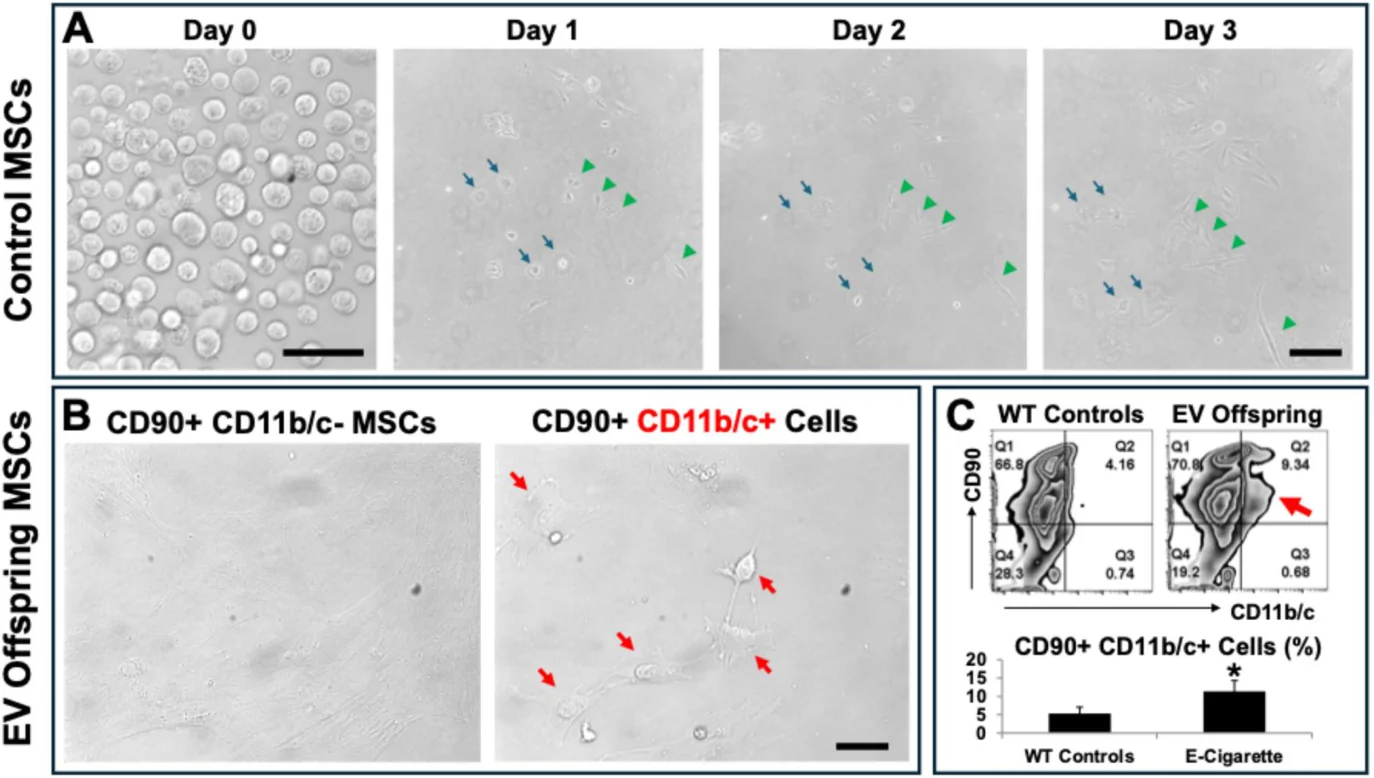
Altered mesenchymal stem/stromal cells (MSCs) in adult offspring bone marrow. (**A**) Representative phase-contrast images showing the morphology of cultured bone marrow-derived MSCs from control rats during the passage procedure from P3 to P4 generation. The time-course study reveals the changes in morphology of floating MSCs (passage 4, P4 with different sizes) and attached MSCs during the next 3 days (from attachment to growth). Two primary MSC morphologies are observed: round-shaped cells (blue arrowheads) and angle-shaped cells (green arrows), highlighting the heterogeneous nature of the MSC population (both floating and attached cells). Scale bar: 50 µm. (**B**) Morphological and immunophenotypic analysis of MSCs from E-cig vaping (EV)-exposed offspring demonstrates two distinct populations: CD90^+^CD11b/c^−^ MSCs (**left panel**), which exhibit a typical spindle-shaped morphology, and CD90^+^CD11b/c^+^ cells (**right panel**, red arrows), which show altered, pro-inflammatory morphology. Scale bar: 10 µm. (**C**) Flow cytometry contour plots and quantification of CD90^+^CD11b/c^+^ cells in bone marrow from wild-type (WT) controls and EV offspring. EV offspring display a significant increase in the CD90^+^CD11b/c^+^ cell population (red arrow, **upper right quadrant**), as shown in the bar graph vs. WT controls. Where applicable, data are presented as means ± SEM. * *p* < 0.05, N = 3 biological replicates.

MSCs are heterogeneous and are known to possess immunomodulatory and reparative properties, which can systemically improve transplantation and tissue regeneration [56]. Our longitudinal in vivo studies demonstrated that there were significantly increased BM MSCs in E-cig-exposed offspring. We then performed in vitro studies to identify further differences in the BM MSCs between E-cig-exposed (EV) and control offspring. In cultures of isolated MSCs from control offspring, there were two routine morphologies: round-shaped and angular-shaped cells, which gradually established their attachments to the dish’s bottom over the following 3-day culture period (blue arrows and green arrowheads, respectively, Figure 3A). However, in cultures of EV MSCs, in addition to the presence of round-shaped and angular-shaped MSCs, there was another emerging population of CD90^+^CD11b/c^+^ cells with altered morphologies (red arrows, Figure 3B). FC and quantitative analyses confirmed a significant increase in CD90^+^CD11b/c^+^ cells in EV offspring compared with the control group (Figure 3C). Consistently, a novel subpopulation of CD34^+^CXCL2^+^ MSCs with low expression of osteoblast markers (e.g., RUNX2) was identified by single-cell RNA-sequencing analysis of heterogeneous MSCs [57], potentially relevant to osteoporosis. Notably, this MSC subset may exist throughout the lifespan and pathologically evolve to support adult oncogenesis [58].

In summary, these findings suggest that maternal vaping during pregnancy may promote the development of a distinct, potentially maladaptive MSC subpopulation in offspring BM, thereby adversely impacting the MSC BM niche.

### 3.6. Maternal Vaping During Pregnancy Promotes Cytokine Dysregulation in Adult Offspring Rats

**Figure 4:**
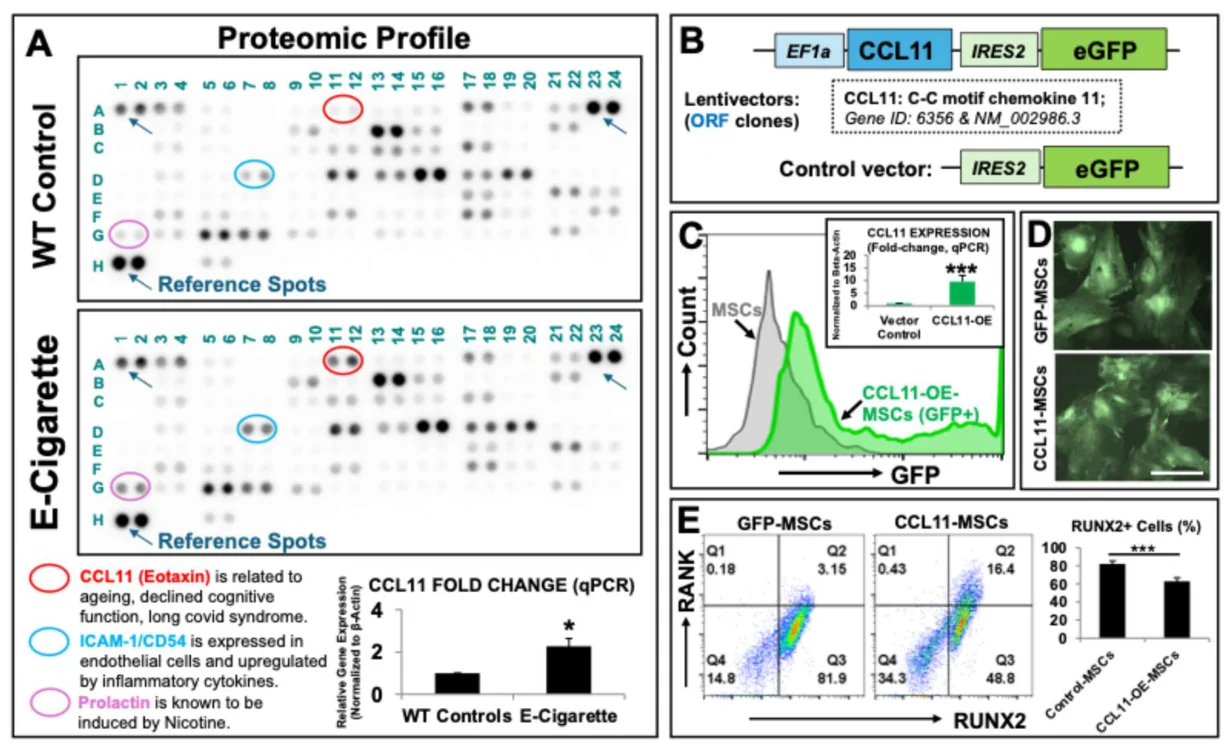
Maternal e-cigarette vaping during pregnancy promotes cytokine dysregulation and impairs osteogenic signaling in adult offspring rats. (**A**) Proteomic profiling of plasma from adult control offspring and in utero E-cig-exposed offspring rats reveals significant upregulation of CCL11 (Eotaxin, red circle), ICAM-1/CD54 (blue circle), and prolactin (purple circle) in the E-cigarette group. Quantitative PCR analysis (bar graph) confirms increased CCL11 mRNA expression in bone marrow of E-cigarette-exposed offspring compared to WT controls. (**B**) Schematic diagram of a lentiviral expression construct containing open-reading frames (ORFs) of the human CCL11 gene and eGFP reporter, with promoters EF1a and IRES2, respectively, which was used to overexpress CCL11 in control (non-EV) MSCs. (**C**) A flow cytometry histogram shows high GFP expression in CCL11-overexpressing MSCs (green) compared to control MSCs without lentiviral transduction (gray). Insert: qPCR assay reveals significantly increased CCL11 gene expression in CCL11-overexpressing MSCs when compared to GFP-positive vector control MSCs. (**D**) Representative fluorescence microscopy images of GFP-positive control MSCs and CCL11-overexpressing MSCs, demonstrating similar morphology. Scale bar: 50 µm. (**E**) Flow cytometry scatter plots (RANK vs. RUNX2) and quantification reveal a significant decrease in RUNX2^+^ osteogenic cells and an increase in RANK^+^ cells in CCL11-overexpressing MSCs compared to controls. Where applicable, data are presented as means ± SEM. * *p* < 0.05, *** *p* < 0.005, N = 3 biological replicates.

Cytokine priming is crucial for self-renewal, lineage differentiation, migration and immune response/modulatory functions of both HSCs and MSCs [59]. To investigate the secretome induced by maternal E-cig vaping, we performed a comprehensive proteomic comparison of plasma samples collected from adult EV offspring rats and controls (a full panel of cytokine changes, Supplementary Figure S1). CCL11 (Eotaxin), an age-related pro-inflammatory cytokine [60], was identified (red circle, Figure 4A) and displayed a significant ~6-fold increase from 2208 to 13,861 integrated density (a sum of pixel values within a defined region of interest) (control group vs. EV offspring). This finding is consistent with marked elevations of CCL11 levels in human smokers [61]. Notably, other aging-associated pro-inflammatory cytokines like IFN-γ, members of the IL-1 family, IL-6, and GDF15, a stress-related mitokine and a key player in the human aging process [62], were also increased in e-cigarette-exposed offspring compared to the control group (Supplementary Figure S1). Additional increases were observed in ICAM-1/CD54 (blue circle), an inflammation-associated adhesion molecule, and prolactin (purple circle), whose plasma increase is known to be a hormonal effect of nicotine exposure in human smokers [63]. qPCR analysis confirmed a 2.3-fold increase in *CCL11* gene expression in the BM of EV offspring compared to controls (bottom right bar graph, Figure 4A), indicating that the BM may have been responsible for systemic CCL11 elevation.

### 3.7. Elevated CCL11 May Impair Osteogenic Signaling of MSCs in Adult EV Offspring

In previous in vitro studies, E-cig aerosol was reported to impair osteogenic differentiation and mineralization of MSCs [64,65]; however, the underlying mechanism was not sufficiently defined. In the present study, FC analysis revealed a significant reduction in FTO^+^ RUNX2^+^ MSCs in EV rat offspring compared to the control group (<20% versus ~68%, Supplementary Figure S2A), suggesting an impaired osteogenic pathway in adult E-cig-exposed offspring MSCs.

Next, to examine the potential role of elevated CCL11 in the osteogenic defect, control (non-EV offspring) MSCs were transduced with a lentiviral CCL11-overexpression (OE) system (Figure 4B). Successful transduction was confirmed by robust GFP expression and qPCR analysis of elevated *CCL11* gene expression (Figure 4C). Morphologically, CCL11-OE cells resembled vector controls (Figure 4D) but displayed significant molecular changes. FC analysis revealed a significant decrease in RUNX2^+^ osteogenic cells and a concomitant increase in RANK^+^ cells (Figure 4E). Consistently, CCL11-OE also significantly suppressed osterix (OSX/Sp7), a key osteoblast maturation factor [66] (Supplementary Figure S2B) and reduced calcified nodule formation compared to controls (Alizarin Red S staining images, Supplementary Figure S2C). In parallel, lentiviral overexpression of FTO gene expression in EV-MSCs increased RUNX2 gene expression compared with controls (Supplementary Figure S2D). Finally, micro-computed tomography (µCT) analysis revealed reduced bone volume fraction (BV/TV) in young adult offspring exposed to maternal vaping (Supplementary Figure S3). These findings support altered osteogenic capacity and bone architecture in adult rat offspring, potentially leading to higher risk of bone fracture and disorders when offspring age [67], but do not by themselves establish osteoporosis, net bone loss, or a complete causal CCL11–FTO–RUNX2 pathway. Consistently, mother rats also showed a clear reduction in bone parameters.

Collectively, these data support an association between elevated CCL11, reduced osteogenic signaling, and altered FTO/RUNX2 expression in the BM niche. The CCL11–FTO–RUNX2 relationship should be considered a candidate regulatory model requiring additional neutralization, loss-of-function, and rescue studies for causal validation.

### 3.8. Nicotine Promotes Myelopoiesis and Pro-Inflammatory Cytokines in Human Peripheral Blood Samples and Cell Line Models Ex Vivo

**Figure 5:**
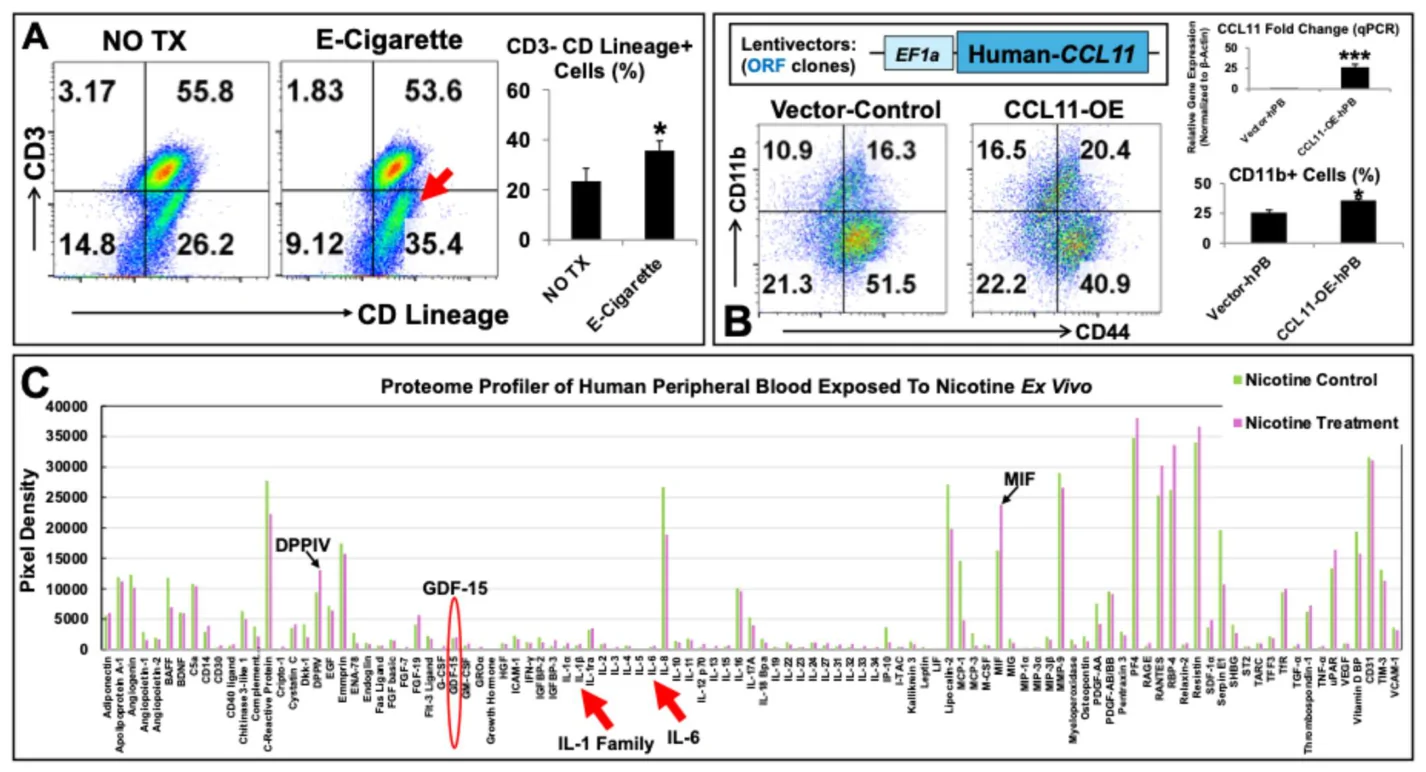
E-cigarette/nicotine exposure is associated with inflammatory and myeloid-associated changes in human peripheral blood (hPB) samples ex vivo. (**A**) Representative flow cytometry plots with a quantitative table show an increased percentage of CD3^−^CD-lineage^+^ cells in hPB samples following ex vivo e-cigarette exposure. (**B**) The human CCL11 gene was lentivirally overexpressed in hPB cells; quantitative gene expression analysis (qPCR) shows upregulation of CCL11 gene expression. Representative flow cytometry plots and quantification display significantly increased CD11b^+^ cells in CCL11-overexpressing (OE) hPB compared to vector control hPB samples ex vivo. (**C**) Cytokine proteomics revealed the significant increase of MIF (macrophage migration inhibitory factor, an important regulator of innate immunity that binds to CD74 on other immune cells to trigger an acute immune response) and DPPIV (dipeptidyl peptidase-4, CD26, which cleaves different peptides, plays roles in various cancers, and is involved in fibrosis of the liver and kidney) (indicated by arrows) in the supernatant of hPB samples that were exposed to 10 μM nicotine treatment ex vivo. Similar to the cytokine proteomics of E-cig-exposed offspring rats, GDF15, a stress signal (indicated by the red circle) and pro-inflammatory cytokines like the IL-1 family and IL-6 (indicated by red arrows) were significantly increased after nicotine treatment in hPB. Where applicable, data are presented as means ± SEM. * *p* < 0.05, *** *p* < 0.005, N = 3 biological replicates.

E-cigarette (nicotine-containing) use, perceived as a safer replacement for traditional tobacco cigarettes, has grown substantially over the last decade. To provide complementary translational evidence, we investigated selected effects of e-cigarette aerosol or nicotine exposure on human immune cells ex vivo using peripheral blood samples from healthy donors (hPB, N = 6). These experiments were designed to determine whether selected inflammatory and myeloid-associated responses observed in the rat model could also be detected in human cells; they were not intended to validate maternal vaping effects in humans. FC analysis revealed an increase in CD3^−^CD lineage^+^ cells from 26.2% to 35.4% in E-cig-exposed hPB samples (Figure 5A). Next, we used the MV4-11, a human acute myeloid leukemia cell line, as a hematologic malignancy model for selected nicotine-exposure and immune co-culture assays. E-cig exposure increased mitotic cells (blue arrows) and induced a 5-fold upregulation of *CCL11* gene expression (Supplementary Figure S4A), with an associated increase in expression of G2-phase cell cycle genes such as a 5.2-fold increase in *CDK1* and a 2.0-fold increase in *Cyclin B1* (*CCNB1*) versus controls (Supplementary Figure S4B). These findings provide ex vivo context for nicotine-associated cellular responses but do not demonstrate that prenatal nicotine exposure causes leukemia or that the offspring marrow phenotype is leukemogenic.

Next, to assess the direct effect of elevated CCL11 on the human blood cells, we genetically overexpressed the human *CCL11* gene in hPB (CCL11-OE-hPB). CCL11 overexpression significantly increased the proportion of CD11b^+^ hPB cells compared to vector controls (Figure 5B). Finally, a comprehensive human cytokine assay of ex vivo nicotine-treated hPB samples revealed significant increases in pro-inflammatory cytokines MIF and DPPIV (CD26), from 16,285 to 23,824 and 9421 to 13,081 integrated density units, respectively (Figure 5C). Notably, consistent with proteomic analysis of E-cig-exposed offspring rats, GDF15, a stress signal, and pro-inflammatory cytokines like the members of the IL-1 family and IL-6 (indicated by the red circle and red arrows, respectively, Figure 5C) were also significantly increased in nicotine-treated human samples. In summary, these ex vivo human studies demonstrate that E-cig/nicotine exposure can induce pro-inflammatory cytokines such as CCL11 and increase CD11b^+^ myeloid-associated cells, providing complementary evidence that selected nicotine-responsive inflammatory and myeloid-associated changes can also be detected in human cells.

### 3.9. Nicotine Impairs Cluster Formation of Human T Cells and Reduces Their Memory and Cytotoxic Function Ex Vivo

T-cell immunity is crucial for targeted defense to maintain health and prevent diseases [68]. In contrast to the rapid turnover of other BM stem cells, T cells are genetically stable and normally long-lived, providing memory protection in mammals [69,70,71]. Accumulating evidence suggests that cancer cells utilize adrenergic neurotransmitters to promote T-cell exhaustion and suppress anti-tumor T-cell responses, thereby reducing the effectiveness of cancer immunotherapies [72,73]. Nicotine is known to hijack the body’s natural adrenergic signaling to promote the progression of vascular disease and cancer [74]. Due to the limited number of studies available, it is important to evaluate whether nicotine can directly alter T-cell immunity and anti-tumor T-cell responses. In our previous study, we reported a cell culture protocol that efficiently isolates and expands primary T cells from adult acute myeloid leukemia (AML) patients (N = 30) [39]. Therefore, in the present study, we investigated how nicotine affects primary human T cells.

**Figure 6:**
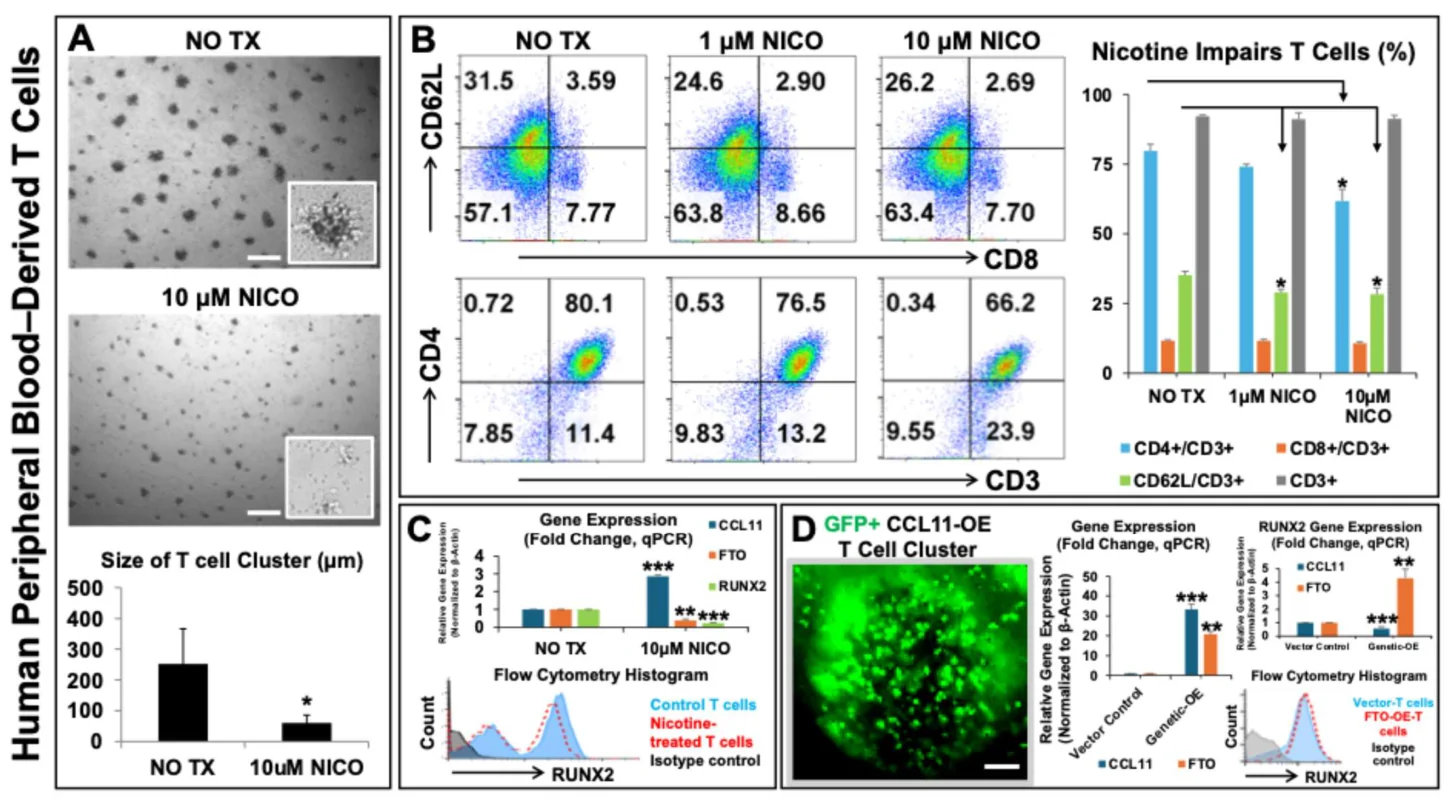
Nicotine exposure alters human T-cell phenotype and CCL11, FTO, and RUNX2 expression ex vivo. (**A**) Representative phase-bright images of T cells isolated from healthy human peripheral blood, which were pretreated with different doses of nicotine; Insets: 20× magnification of T-cell clusters; Lower panel: Cumulative size data of T-cell clusters in different experimental groups. Scale bar: 500 µm. (**B**) Representative flow cytometry plots with a quantitative table show CD3^+^, CD62L^+^, CD4^+^, CD8^+^ expression in different experimental groups of nicotine treatment. (**C**) Upper panel: qPCR analysis of CCL11, FTO, and RUNX2 gene expression in the experimental group of 10 μM nicotine treatment. Lower panel: flow cytometry histogram reveals the reduced RUNX2 protein expression in the experimental group of 10 μM nicotine treatment when compared to the control group. (**D**) Representative fluorescence microscopy image of a GFP-positive CCL11-overexpressing (OE) human T-cell cluster. Scale bar: 100 µm. Middle bar graph: qPCR analysis of CCL11 and FTO gene expression in human T cells overexpressing CCL11 or FTO transgenes. Right upper bar graph: qPCR analysis of RUNX2 gene changes in human T cells overexpressing CCL11 or FTO transgenes. Right lower panel: flow cytometry histogram reveals that FTO-OE significantly increases RUNX2 protein expression compared to the vector-control group. Where applicable, data are presented as means ± SEM. * *p* < 0.05, ** *p* < 0.01, *** *p* < 0.005, N = 3 biological replicates.

First, we isolated and expanded T cells from healthy adult peripheral blood (hPB) samples (N = 6). We then found that nicotine (10 µM) treatment for 48 h significantly reduced the size of T-cell clusters, a recognized biomarker of proliferation [39] (Figure 6A). Consistently, FC analysis showed that nicotine treatment significantly reduced the percentage of CD62L^+^ memory T cells (Figure 6B), which also expressed CCR7+, a biomarker of naïve T cells (Supplementary Figure S5A). Additionally, FC analysis showed that nicotine (10 µM) treatment significantly reduced the percentage of CD4^+^ T cells without affecting the percentages of CD3^+^ and CD8^+^ T cells (Figure 6B). Consistently, treatment with nicotine or tobacco extract has also been reported to decrease the percentage of murine CD4^+^ T cells in vitro [75].

Next, to examine whether selected CCL11-, FTO-, and RUNX2-associated changes identified in the experimental framework could also be detected in human T cells, we performed qPCR analysis and found that nicotine (10 µM) treatment significantly elevated the gene expression of CCL11 while reducing the gene expression of FTO (essential for effective CD4^+^ T-cell immune response [76]) and RUNX2 (required for long-term maintenance of memory CD8^+^ T cells [77]) compared with control T cells (Figure 6C). Furthermore, we employed lentiviral vectors to overexpress CCL11 and FTO transgenes in human T cells. qPCR analysis confirmed that CCL11 overexpression (OE) significantly suppressed RUNX2 gene expression, whereas FTO-OE markedly increased RUNX2 gene and protein expressions in T cells (Figure 6D).

Finally, to evaluate anti-leukemia activity under the specific experimental conditions used, nicotine-treated T cells were co-cultured with allogeneic MV4-11 AML cells. Nicotine (10 µM)-treated T cells displayed a reduced anti-leukemia effect compared with control T cells, as evidenced by a significantly increased number of viable CD15^+^ blasts (Supplementary Figure S5B). Mechanistically, Seahorse analysis indicated altered cellular metabolism in T cells after nicotine exposure (Supplementary Figure S5C). Increasing evidence indicates that nicotine and e-cigarette exposure disrupt the mitochondrial transcriptome and compromise mitochondrial function [78,79], which is consistent with our transcriptomic analysis showing the reduced gene expression of key transcriptional regulators of mitochondrial biogenesis in E-cig-exposed offspring BM cells (Figure 2H). Resultant mitochondrial dysfunction forces T cells to rely on glycolysis, heightening their dependence on extracellular glucose. However, AML blasts preferentially consume glucose via the Warburg effect, thereby metabolically outcompeting T cells. This glucose restriction limits T-cell proliferation and cytotoxic function, promoting leukemia progression [80].

In summary, the primary human T-cell studies indicated that nicotine exposure altered T-cell proliferation and memory-associated phenotype and was associated with reduced anti-leukemia activity in a proof-of-principle co-culture assay ex vivo. Changes in CCL11, FTO, and RUNX2 expression and the effects of CCL11/FTO overexpression support a candidate regulatory relationship. However, because degranulation, intracellular cytokine production, molecule expression, and immune checkpoint profiling of nicotine-altered T cells were not comprehensively assessed, the present experiments do not establish a complete causal CCL11–FTO/RUNX2 regulatory axis in human T cells.

### 3.10. E-Cigarette-Exposed Offspring-Derived BM CD11b/c^+^ Cells Are Associated with Impaired T-Cell Growth and Increased PD-L1 Expression In Vitro

Tumor-associated macrophages (TAMs) in the tumor microenvironment (TME) constitute a major immunosuppressive barrier (both physically and metabolically) to effective immunotherapies (e.g., CAR-T cell therapies) and facilitate tumor progression [81]. To evaluate how maternal E-cig vaping during pregnancy affected T-cell and myeloid-cell interactions in offspring BM, we performed in vitro cultures (using the same T-cell culture system) of BM cells isolated from control offspring and E-cig-exposed offspring rats (postnatal day 10). There was a significant increase in T-cell growth, in terms of both cluster number and size, in control offspring compared with E-cig-exposed offspring rats (Supplementary Figure S6A). In contrast, there was a significantly increased number of CD11b/c^+^ cells with macrophage-like morphology [82] attached to the bottom of the culture dish in E-cig-exposed offspring compared with control offspring rats (Supplementary Figure S6B). The rapid in vitro expansion of CD11b/c^+^ cells is consistent with our in vivo flow cytometry results showing a significant increase in CD45^+^ cells in BM tissues of P7 E-cig-exposed offspring rats compared with controls (Figure 1F). Notably, a significant elevation in programmed death-ligand 1 (PD-L1) expression in the CD11b/c^+^ compartment from E-cig-exposed offspring (Supplementary Figure S6B) indicates that a maternal E-cig (nicotine)-induced myeloid-enriched compartment may have functional immunosuppressive consequences on offspring T-cell growth (Supplementary Figure S6A). Consistently, prior evidence has shown that lung cancer patients who smoke tobacco generally have a higher PD-L1 tumor proportion score [83].

Altogether, these findings provide proof-of-principle evidence that the altered CD11b/c^+^ myeloid-enriched compartment in E-cigarette-exposed offspring BM is associated with increased PD-L1 expression and reduced T-cell growth in vitro. Because CD11b/c is a broad myeloid marker, the specific subset of CD11b/c^+^ myeloid-associated cells responsible for this effect remains to be determined.

## 4. Discussion

**Figure 7:**
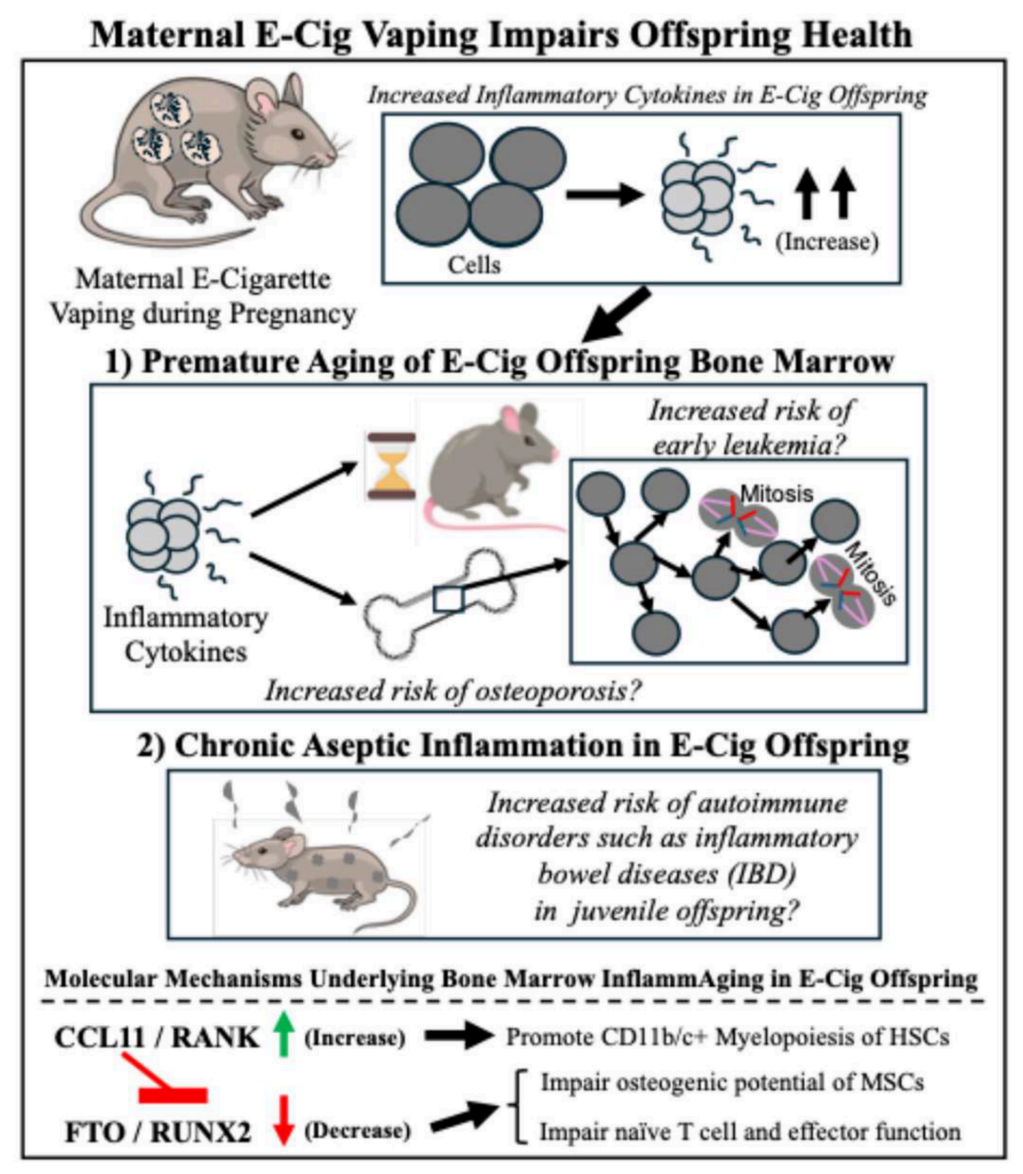
Overview of long-term impact of maternal e-cigarette exposure during pregnancy on offspring health. The schematic summarizes the observed associations with inflammatory and aging-associated cytokines, expansion of the CD11b/c^+^ myeloid-enriched compartment, altered T-cell phenotype/function, and reduced osteogenic potential in rat offspring BM. Mechanistically, our findings suggest CCL11, FTO, and RUNX2 as a candidate regulatory network rather than a definitively established causal pathway. CCL11 may act as an inflammaging-associated niche signal that coordinately suppresses FTO-dependent RNA stability and RUNX2-driven differentiation in mesenchymal stem cells and immune cells.

In the present study, our longitudinal evidence spanning in vivo rat studies, complementary human primary cell experiments, and mechanistic in vitro assays demonstrates persistent associations between maternal e-cigarette exposure and altered hematopoietic, stromal, inflammatory, transcriptional, and osteogenic phenotypes in offspring (Figure 7). These findings provide a framework for investigating how developmental exposure may influence later-life disease susceptibility in offspring.

### 4.1. Hematopoietic Stem Cells (HSCs) and Myelopoiesis: Developmental Reprogramming Toward an Aged Hematopoietic State

The collective findings support developmental alterations in immune and hematopoietic homeostasis in rat offspring BM following maternal e-cigarette exposure. Expansion of the CD11b/c^+^ myeloid-enriched compartment, inflammatory cytokine changes, and transcriptomic enrichment of aging-associated pathways are consistent with an aging-associated inflammatory state. However, these findings do not by themselves demonstrate cellular senescence or premature HSC aging. Direct assessment of senescence markers, DNA damage, telomere status, and functional HSC self-renewal would be required to establish these processes definitively.

One of the earliest manifestations of this phenotype is splenomegaly (Figure 1E), which often reflects pathological extramedullary hematopoiesis. Under normal conditions, hematopoiesis is restricted to the bone marrow after birth; however, prenatal nicotine exposure may cause aberrant seeding and proliferation of HSC progenitors in the spleen. The enlarged spleens observed in vaping offspring therefore suggest functional impairment of the bone marrow microenvironment and represent an anatomical marker of hematopoietic niche dysfunction.

A central hallmark of hematopoietic aging is myeloid skewing, characterized by preferential differentiation of HSCs toward myeloid lineages at the expense of lymphoid output [84]. Vaping offspring exhibited expansion of CD11b/c^+^ myeloid populations and a pro-inflammatory state, closely resembling the aged HSC phenotype described in elderly animals and humans. This shift is particularly significant because myeloid bias is considered one of the most robust biomarkers of biological aging and correlates with DNA methylation-based aging clocks [85]. Importantly, aging-associated expansion of the myeloid compartment does not indicate improved innate immunity such as phagocytosis or antigen presentation but may represent a failure of cell death coordination [86].

The myeloid phenotype is accompanied by a systemic inflammatory profile that includes increased IL-1-family cytokines, IL-6, CCL11 [87,88,89], and GDF-15 [62], potential biomarkers for aging prediction algorithms. These findings are consistent with chronic low-grade inflammation and an inflammaging-associated molecular environment. However, because canonical senescence assays were not performed, the present data should be interpreted as inflammatory and aging-associated signatures rather than direct evidence of a senescence-associated secretory phenotype (SASP).

### 4.2. Mesenchymal Stem Cells (MSCs) and Premature Osteogenic Alterations: Molecular Dysregulation of the FTO-RUNX2 Pathway

Maternal e-cigarette exposure during pregnancy was associated with significant changes in the stromal/mesenchymal compartment and impaired osteogenic differentiation in rat offspring BM. These findings are compatible with altered stromal function rather than tissue repair [90]. E-cig-exposed offspring MSCs may actively contribute to bone marrow aging through secretion of IL-1, IL-6, TNF-α, and CCL11. These inflammatory mediators promote myeloid differentiation, suppress lymphopoiesis, reduce HSC quiescence, and impair osteogenic differentiation, creating a self-reinforcing cycle of inflammation and tissue degeneration; however, the present study does not establish a complete causal inflammatory feedback loop.

A notable observation was the emergence of a CD90^+^/CD11b/c^+^ hybrid stromal–myeloid population within the bone marrow niche, absent in controls. This population may represent either myeloid-derived suppressor cells acquiring stromal characteristics or MSCs undergoing inflammatory lineage reprogramming toward fibrocyte-like or myofibroblast-like states. Because CD90 and CD11b/c are insufficient to define a unique MSC lineage, this population may also represent heterogeneous stromal or myeloid-associated cells. Previous single-cell transcriptomic analyses revealed a similar CXCL2-expressing MSC subpopulation characterized by low RUNX2 expression and impaired osteogenic potential [57]. However, multiparameter phenotyping and lineage-resolved studies will be required to determine its precise identity and functional contribution.

Maternal vaping was also associated with reduced bone volume fraction (BV/TV) in young adult offspring (Supplementary Figure S3). Together with reduced RUNX2/Osterix expression and impaired mineralization demonstrated by Alizarin Red staining, these findings support altered osteogenic potential and bone architecture. However, the present study did not comprehensively measure osteoblast/osteoclast markers or dynamic bone histomorphometry; therefore, conclusions regarding net bone resorption, osteoporosis, or future fracture risk should be considered preliminary. The FTO–RUNX2 relationship is supported by reduced FTO expression and the observed increase in RUNX2 following FTO overexpression (Supplementary Figure S2), but direct m^6^A mapping and FTO loss-of-function studies are needed to establish causality.

In summary, maternal vaping was associated with impaired offspring osteogenic potential and altered FTO/RUNX2 signaling, with potential implications for long-term skeletal health. These findings support a candidate epitranscriptomic mechanism that warrants further functional validation rather than establishing a complete FTO–RUNX2 pathway or a premature osteoporosis phenotype.

### 4.3. Significance of KLF4-Notch 1-FTO Dysregulation in E-Cig Offspring Bone Marrow

Our transcriptomic analyses demonstrated broad changes in transcriptional activity and molecular networks in E-cigarette-exposed offspring BM cells (Figure 2E–G). These alterations were accompanied by increased KLF4 expression, decreased NOTCH1 and FTO expression, expansion of the CD11b/c^+^ myeloid-enriched compartment, impaired osteogenic differentiation, and altered human T-cell function. The convergence of these phenotypes suggests persistent developmental reprogramming toward an inflammatory and aging-associated molecular state; however, these associations do not establish a premature-aging phenotype or a single causal molecular switch.

Mechanistically, KLF4 and NOTCH1 are central regulators of stem cell fate decisions, tissue regeneration, and immune development [28]. KLF4 is a key pluripotency factor that regulates HSC self-renewal and lineage commitment [27], whereas NOTCH1 signaling maintains HSC homeostasis, osteogenic differentiation, and T-cell lineage specification [91,92]. KLF4 has been reported to promote SASP and inflammatory signaling [93]. Therefore, the coordinated increase in KLF4 and suppression of NOTCH1 observed in nicotine-exposed offspring suggests the existence of a pathogenic KLF4–NOTCH1 imbalance [94,95] that may have contributed to the coordinated inflammatory, hematopoietic, and osteogenic alterations observed in this experimental model. Importantly, this mechanism provides a biologically plausible explanation for how prenatal nicotine exposure can simultaneously affect HSCs, MSCs, and immune cell development through disruption of shared developmental signaling networks in the E-cig offspring bone marrow.

Growing evidence indicates that m^6^A modifications are critical regulators of HSC lineage commitment [96] and T-cell homeostasis [97]. Thus, suppression of FTO, the m^6^A demethylase, may contribute to the widespread transcriptional repression observed in nicotine-exposed offspring by altering RNA processing and gene expression programs that govern stem cell maintenance and immune competence. The simultaneous alterations in KLF4–NOTCH1-associated developmental signaling and FTO expression suggest a candidate integrated regulatory framework that may contribute to persistent developmental reprogramming and an inflammation- and aging-associated molecular state after prenatal e-cigarette exposure. Moreover, RNA-sequencing analysis of human T cells overexpressing FTO demonstrated that FTO overexpression may partially modify molecular programs altered in nicotine-exposed human T cells by promoting the overall gene expression, including cytokine pathways and the CMG helicase complex for T-cell proliferation and DNA replication (Supplementary Figure S7). Additionally, ectopic FTO overexpression can promote the gene expressions of TFAM, an essential transcription factor responsible for mitochondrial biogenesis and ATP energy production [41] and suppress PDCD1 (encoding PD-1, a critical immune checkpoint receptor for PD-L1) in FTO-engineered human T cells, representing a potentially applicable strategy for enhancing T-cell metabolic fitness and therapeutic efficacy in regenerative medicine and cancer immunotherapy.

### 4.4. Study Limitations

Several limitations should be considered. First, the present study examined directly exposed F1 rat offspring and therefore does not address potential multigenerational or transgenerational effects. Whether the molecular and mitochondrial alterations observed in F1 offspring persist in subsequent generations and contribute to sustained bone marrow stem cell dysfunction warrants further investigation using F2 and F3 offspring. In addition, larger, prospectively powered cohorts with sex incorporated as a prespecified biological variable are needed to validate these findings and more clearly define potential sex-specific effects. Second, the transcriptomic cohort was relatively small and based on bulk BM tissue, limiting statistical power and cell-type-specific resolution. Future studies should incorporate larger cohorts together with single-cell and/or spatial transcriptomic approaches. Third, the present study did not include direct measurements of cellular senescence within defined BM stem/progenitor cell compartments, such as SA-β-gal, p16INK4a, p21Cip1, p53, γH2AX, Lamin B1, telomere-associated measurements, or functional assessment of HSC self-renewal through serial transplantation. Accordingly, the observed findings should be interpreted as an inflammation- and aging-associated molecular phenotype rather than evidence of direct cellular senescence. Fourth, CD11b/c represents a heterogeneous myeloid marker, and the CD90^+^CD11b/c^+^ population cannot be definitively assigned to an MSC subset without expanded phenotypic characterization and lineage-resolved analyses. Fifth, additional markers of osteogenic and osteoclastic activity and dynamic bone histomorphometry were not assessed and will be important for more comprehensively defining the skeletal consequences of prenatal E-cig exposure. Sixth, although our findings identify associations among CCL11, FTO, and RUNX2, establishment of a causal CCL11–FTO–RUNX2 pathway and determination of CCL11–CCR3 dependence will require receptor-blockade and loss-of-function studies, direct m^6^A mapping, and appropriate rescue experiments. Seventh, the human-cell studies were complementary ex vivo experiments designed to provide mechanistic and translational context and do not establish the clinical effects of maternal E-cig vaping in humans. Larger donor cohorts and broader functional characterization of human immune-cell populations will be required to determine the relevance of these observations to human exposure.

The timing of exposure represents an additional consideration. Extensive epigenetic reprogramming occurs during mammalian preimplantation development, with progressive establishment of lineage-specific chromatin and DNA methylation landscapes during subsequent embryonic development [98,99]. In the present study, chronic intermittent E-cigarette exposure (CIEC) was initiated at embryonic day 4 (E4), thereby modeling in utero exposure beginning during early embryonic development rather than preconceptional or peri-conceptional exposure. Consequently, our model does not address potential effects of maternal E-cig use before conception or during the earliest stages following fertilization. Future studies extending exposure into the preconceptional and peri-conceptional periods will be important for determining whether the timing of maternal exposure differentially influences developmental and epigenetic programming.

Finally, our experiments were conducted using BluPlus E-cigarettes containing nicotine and therefore may not capture the diversity of currently available E-cigarette products, including nicotine-free formulations or products with different solvents, flavoring chemicals, thermal degradation products, and metal constituents [100]. Moreover, aerosol particle concentration and composition, specific chemical constituents, and the individually deposited nicotine dose were not directly quantified in the present study. These factors limit extrapolation of our findings to other E-cigarette devices, formulations, and exposure conditions. Future studies incorporating detailed aerosol characterization, internal nicotine/cotinine dosimetry, and additional E-cigarette products and formulations will be important for defining exposure–response relationships and strengthening the relevance of these findings to public health.

## 5. Conclusions

Our study provides evidence that maternal e-cigarette vaping during pregnancy was associated with persistent hematopoietic, inflammatory, stromal, transcriptional, and osteogenic alterations in the bone marrow of rat offspring. These findings raise the possibility that prenatally induced molecular and epigenetic alterations may contribute to the persistent reprogramming of bone marrow stem cells and their niche, thereby potentially affecting offspring health across the lifespan (Figure 7). Further studies are needed to define the causal mechanisms underlying these alterations, determine their relevance to maternal e-cigarette use in humans, and identify potential therapeutic strategies for preventing and reversing persistent defects and restoring bone marrow homeostasis.

## Data Availability

All original datasets are presented in this article and Supplementary Materials.

## Supplementary Materials

The following supporting information can be downloaded at: https://www.mdpi.com/article/10.3390/cells15171521/s1, Figure S1: Proteomic profiling of cytokines from adult control offspring and in utero E-cig-exposed offspring rats in PB; Figure S2: CCL11-overexpression (OE) suppresses Osterix (OSX, Sp7), a key transcription factor for osteoblast maturation in vitro; Figure S3. Maternal e-cigarette vaping during pregnancy may promote bone disorders in adult offspring; Figure S4. E-cigarette (nicotine) exposure promotes the proliferation of human cell lines through accelerated cell cycle progression ex vivo; Figure S5. Nicotine impairs human peripheral blood-derived T cells and reduces their anti-leukemic blast function ex vivo; Figure S6: E-cig-exposed offspring-derived bone marrow CD11b/c^+^ cells impair T cell growth through increased PD-L1 expression in vitro; Figure S7. RNA-sequencing analysis of genetically engineered human T cells overexpressing FTO (FTO-OE) compared with control human T cells (Vector Control). Table S1: List of reagents used in this study; Table S2: List of Primers (human, OriGene) used in this study.

## Abbreviations

AML: acute myeloid leukemia; BM: bone marrow; CCL11: C–C motif chemokine 11; CIEC: chronic intermittent e-cigarette device; E-cigs: e-cigarettes; FC: flow cytometry; FTO: fat mass and obesity-associated protein; HSC: hematopoietic stem cell; m^6^A: N6-methyladenosine; MSC: mesenchymal stem cell; OSX: osterix; P7: postnatal day 7; PB: peripheral blood; PD-L1: programmed death-ligand 1; RANK: receptor activator of nuclear factor κ B; RUNX2: runt-related transcription factor 2; TAMs: tumor-associated macrophages; TME: tumor microenvironment; μCT: micro-computed tomography.
