# Supplementary material for: Maternal E-Cigarette Vaping Drives Persistent Reprogramming of Bone Marrow Hematopoietic and Mesenchymal Stem Cells and Promotes Transcriptional and Metabolic Dysregulation-Associated Inflammaging and Disease Risks in Rat Offspring
Source: Cells. 2026 Aug 24;15(17):1521. doi: 10.3390/cells15171521 (PMC13565029; doi:10.3390/cells15171521)
Supplement: Supplementary file 1 [file cells-15-01521-s001.zip › cells-4455467-supplementary.pdf]

cells

MDPI

Supplementary Materials:

Flow Cytometry Methodology

We expanded the Supplementary Methods to describe the sequential gating strategy and control procedures used in the study. Briefly, cellular events were initially identified according to forward- and side-scatter characteristics, followed by exclusion of debris and doublets to define the singlet-cell population. Marker-positive populations were subsequently quantified within the appropriate parent populations using the indicated antibody panels. Gates were established using corresponding unstained/isotype and fluorescence-minus-one (FMO) controls, as applicable, and fluorescence compensation was performed for multicolor experiments before analysis in FlowJo. The general flow cytometry acquisition and analysis methodology has also been described in our previous publications (Ref#45: Cao et al., Biomedicines 10 (2022), PMID: 35625776, and Ref#46: Xu et al., Translational Oncology, 2020; 13:100869; PMID: 32956997), which are cited as additional methodological references. Representative flow-cytometry plots are provided throughout the main and supplementary figures.

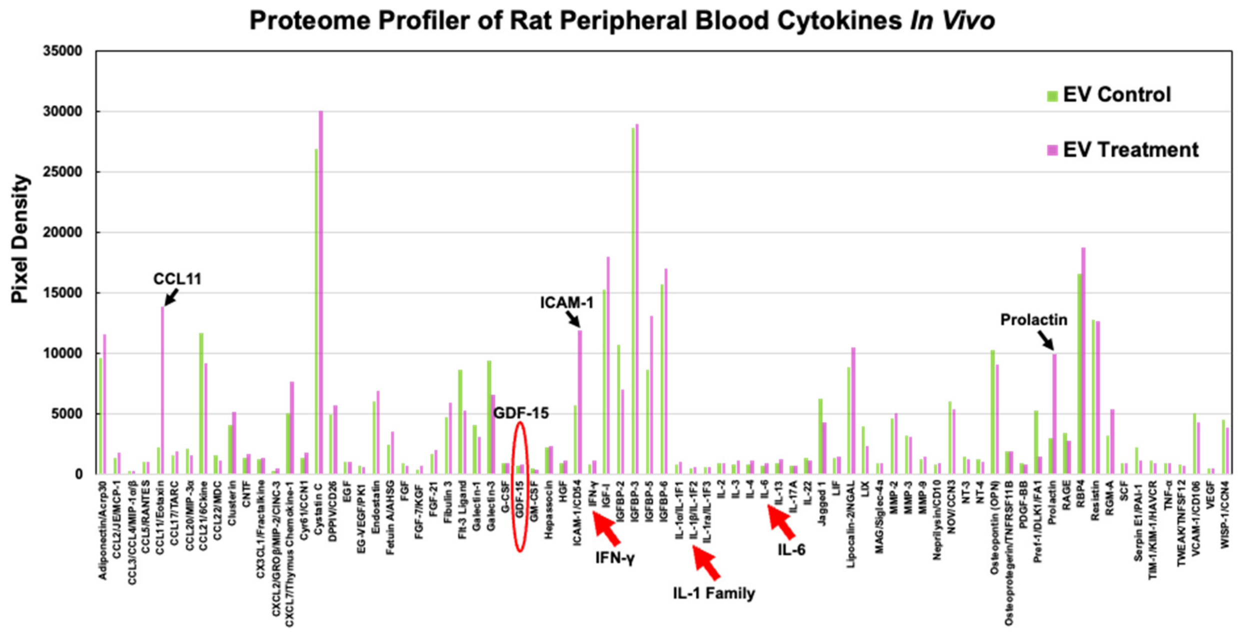

Supplementary Figure S1: Proteomic profiling of cytokines from adult control offspring and *in utero* E-cig-exposed offspring rats in PB.

GDF15, a stress signal (indicated by a red circle) and pro-inflammatory cytokines like the IL-1 family and IL-6 (indicated by red arrows) were significantly increased in e-cigarette-exposed offspring.

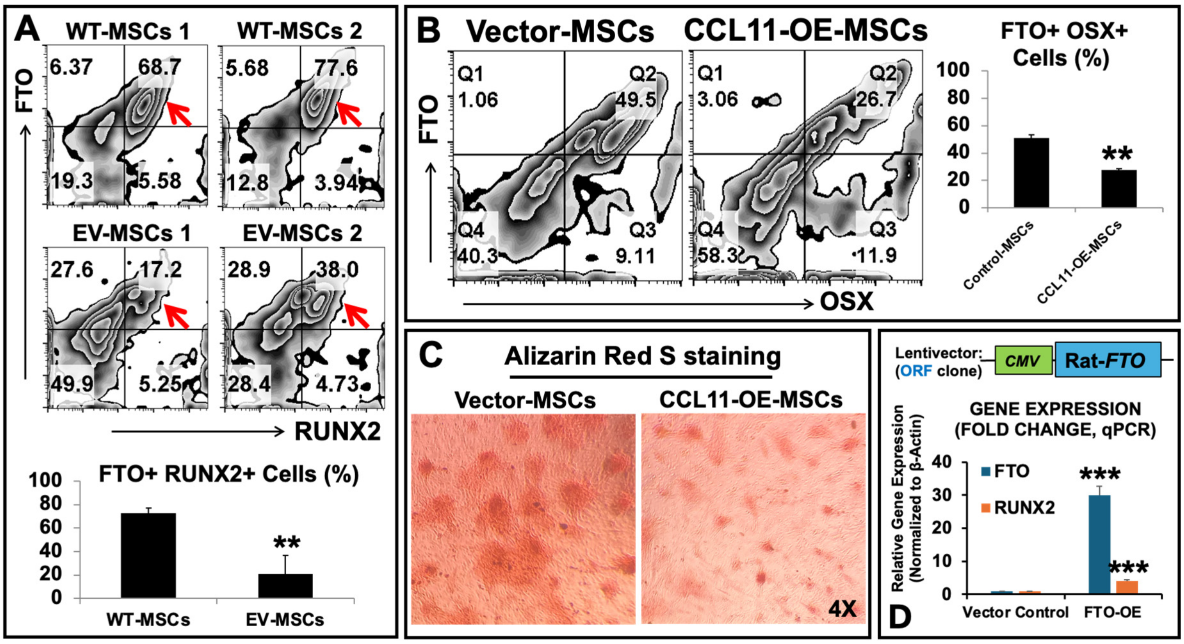

Supplementary Figure S2: CCL11-overexpression (OE) suppresses Osterix (OSX, Sp7), a key transcription factor for osteoblast maturation in vitro.

A) Representative flow cytometry plots show co-expression of FTO and RUNX2 in wild-type MSCs (WT-MSCs, top panels) and *in utero* E-cig-exposed offspring MSCs (EV-MSCs, bottom panels). Red arrows highlight the FTO$^+$RUNX2$^+$ cell populations. Quantification (bar graph, lower) demonstrates a significant reduction in the percentage of FTO$^+$RUNX2$^+$ cells in EV-MSCs compared to WT-MSCs.

B) Representative flow cytometry plots show the expression of FTO and OSX in Vector-MSCs (control) and CCL11-OE-MSCs; quantitative bar graphs demonstrate a significant reduction in the percentage of FTO$^+$OSX$^+$ cells in CCL11-OE-MSCs compared to control-MSCs.

C) Representative Alizarin Red S staining images (4× magnification) display reduced calcified nodule formation in CCL11-OE-MSCs compared to controls.

D) Rat FTO gene with a CMV promoter was lentivirally overexpressed in EV-offspring MSCs; quantitative gene expression analysis (qPCR) shows upregulation of FTO and RUNX2 in FTO-overexpressing EV-MSCs compared to vector controls. Furthermore, consistent with qPCR analysis, the study of ectopic overexpression of the FTO transgene (GeneCopoeia, Cat#: LPP-Rn18290-Lv105-A00) with flow cytometry analysis also confirmed the specificity of the FTO antibody (Cell Signaling Technology, Cat#: 45980), which displays robust staining of the cells overexpressing FTO proteins (not shown).

Where applicable, data are presented as means ± SEM. $^{**}p < 0.01$, $^{***}p < 0.005$, N = 3 biological replicates.

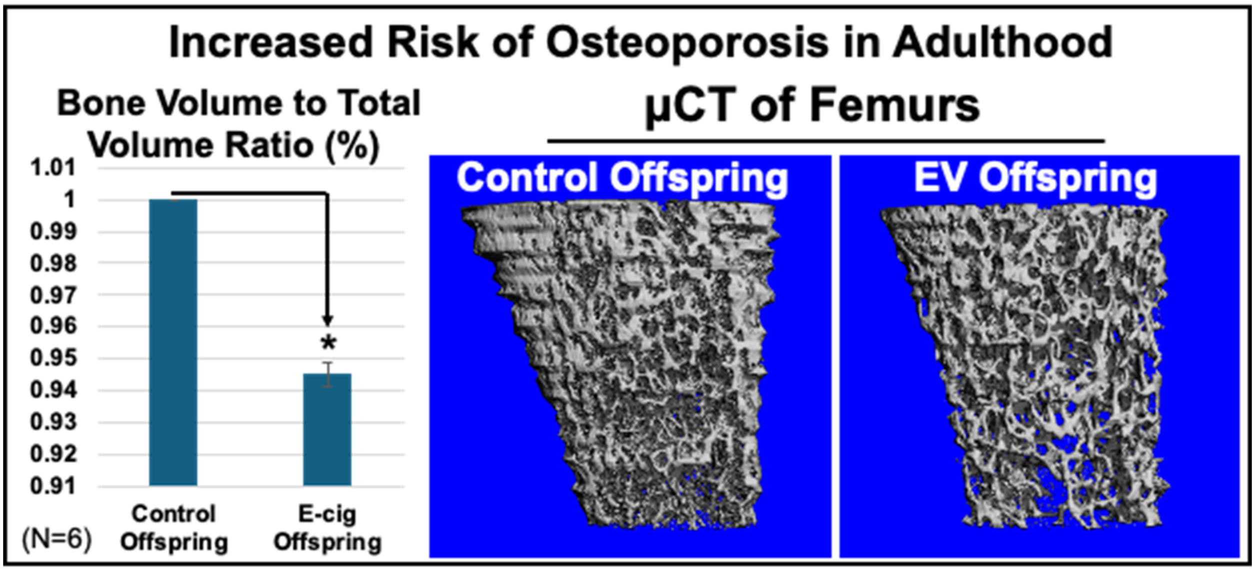

Supplementary Figure S3. Maternal e-cigarette vaping during pregnancy may promote bone disorders in adult offspring.

Micro-computed tomography (μCT) scanning and quantitative X-ray analyses reveal a significantly reduced ratio of Bone Volume to Total Volume (BV/TV) in femoral bones of EV-exposed young adult offspring when compared with control offspring (4-6 weeks old, N=6). **Right:** μCT 3D images of femoral bones indicate the risk of developing osteoporosis and fragile bones in adulthood.

Where applicable, data are presented as means ± SEM. $*p < 0.05$. N = 6 biological replicates.

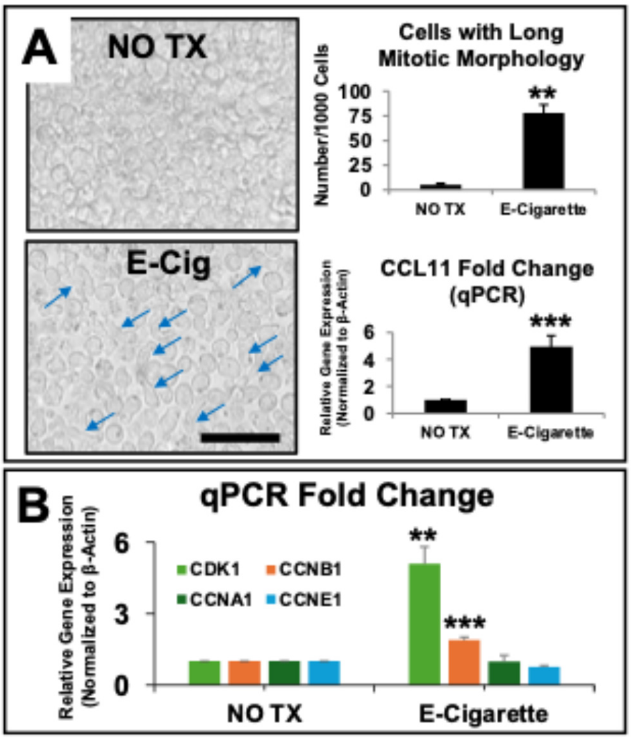

Supplementary Figure S4. E-cigarette (nicotine) exposure promotes the proliferation of human cell lines through accelerated cell cycle progression ex vivo.

A) Representative images showing increased mitotic proliferation of MV4-11, a human AML cell line following nicotine exposure *ex vivo*. Mechanistically, E-cigs promoted the mitotic proliferation of MV4-11 through significantly upregulating the gene expression of pro-inflammatory CCL11 *in vitro*. Scale bar: 50μm.

B) qPCR analyses of cell cycle progression revealed the increased gene expressions of CDK1 and CYCLIN B1 in MV4-11 cells.

Where applicable, data are presented as means ± SEM. **p* < 0.05, ***p* < 0.01, ****p* < 0.005, N = 3 biological replicates.

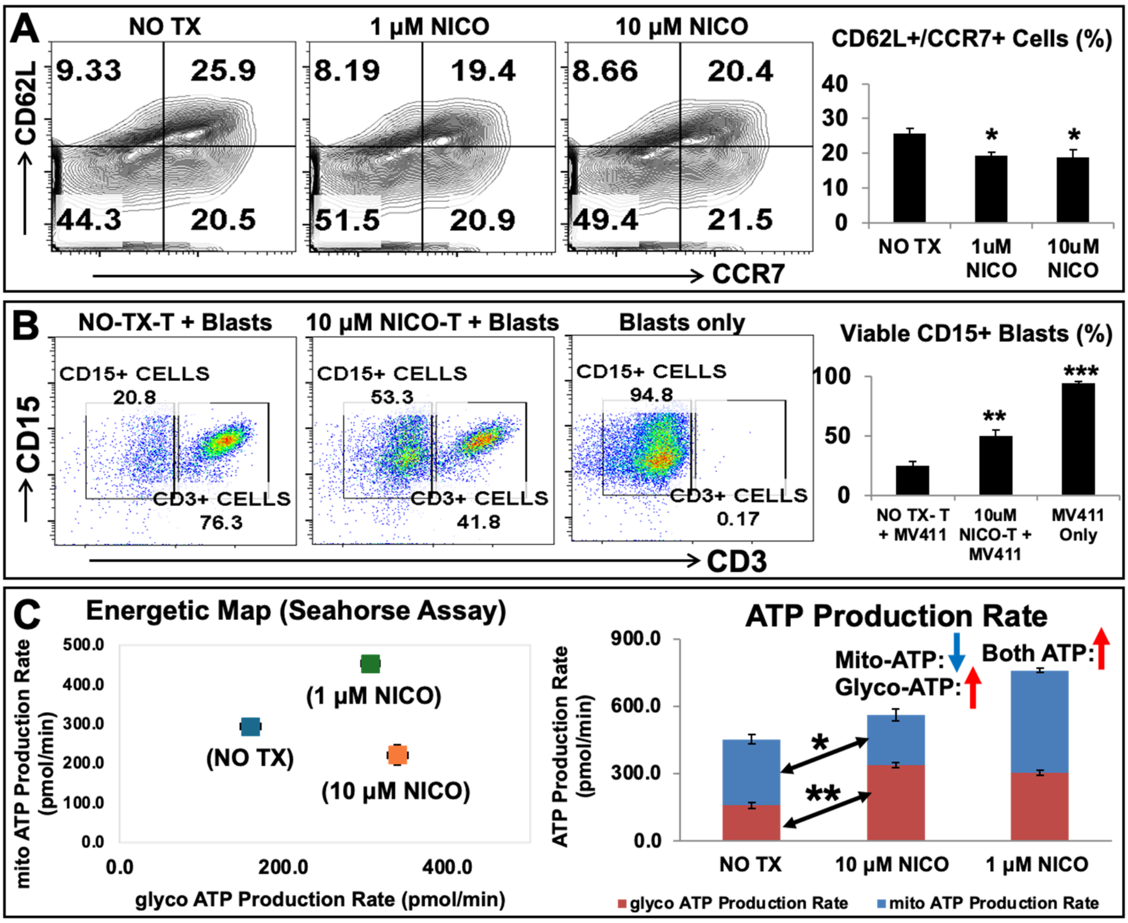

Supplementary Figure S5. Nicotine impairs human peripheral blood-derived T cells and reduces their anti-leukemic blast function ex vivo.

A) FC plots and quantitative bar graphs show significantly reduced CD62L^{+}CCR7^{+} T cells in the presence of nicotine concentrations of 1 μM and 10 μM.

B) Representative FC plots with a quantitative table show that nicotine (10 μM)-treated T cells displayed inferior anti-leukemia cytotoxic function when compared to control (NO-TX) T cells *in vitro*.

C) Seahorse assays show that 10 μM nicotine-treated T cells displayed impaired mitochondrial ATP production which forced T cells to increase glycolysis (an inefficient way) to produce ATP.

Where applicable, data are presented as means ± SEM. **p* < 0.05, ***p* < 0.01, ****p* < 0.005, N = 3 biological replicates.

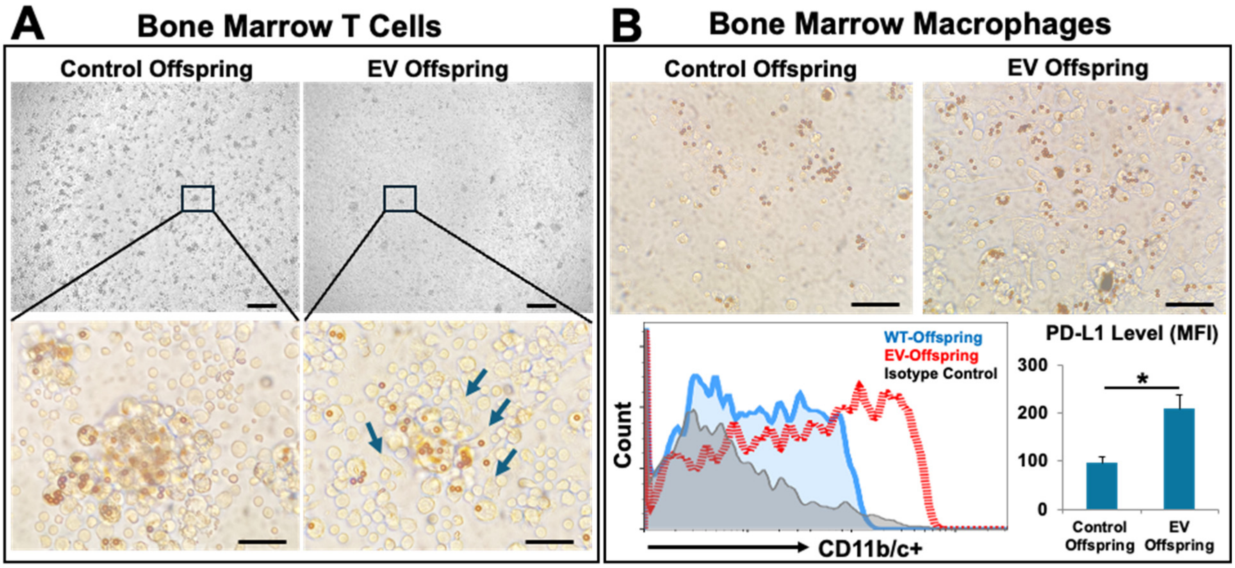

Supplementary Figure S6: E-cig-exposed offspring-derived bone marrow CD11b/c⁺ cells impair T cell growth through increased PD-L1 expression in vitro.

A) Upper panels: Representative phase-bright images of T cell clusters cultured from bone marrow (BM) tissues of control offspring and E-cig-exposed offspring rats (age: P10). There was a significant increase in T cell growth, in terms of both cluster number and size, in control offspring compared with E-cig-exposed offspring rats. Scale bar: 1mm.

Lower panels: Higher-magnification images of T cell clusters; Arrows indicate an increased number of macrophage-like myeloid cells attached to the bottom of the culture dish in E-cig-exposed offspring compared with control offspring rats. Scale bar: 50 µm.

B) Upper panels: Representative phase-bright images of macrophage-like myeloid cells cultured from the BM tissues of control offspring and E-cig-exposed offspring rats (age: P10); There were more macrophage-like myeloid cells in E-cig-exposed offspring rats than in the control group; Scale bar: 50µm.

Lower panels: Flow cytometry histogram confirmed a significant increase in CD11b/c⁺ cells cultured from the BM tissues of E-cig-exposed offspring compared with control offspring rats. Additionally, cumulative data (right panel) of mean fluorescence intensity (MFI) levels of programmed death-ligand 1 (PD-L1) expression revealed a significant increase in PD-L1 expression in CD11b/c⁺ cells cultured from the BM tissues of E-cig-exposed offspring compared with the control group.

Where applicable, data are presented as means ± SEM. *$p < 0.05$, N = 3 biological replicates.

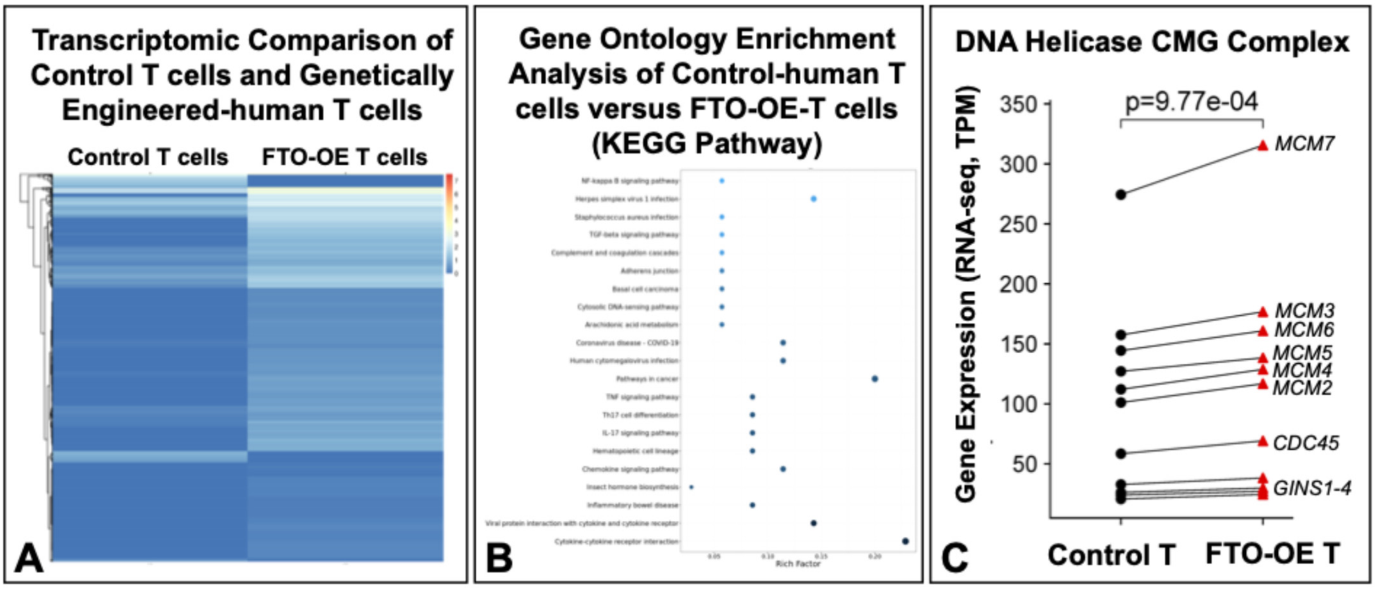

Supplementary Figure S7. RNA-sequencing analysis of genetically engineered human T cells overexpressing FTO (FTO-OE) compared with control human T cells (Vector Control).

A) Transcriptomic profiling shows that FTO overexpression increased global gene expression in human T cells, consistent with the established role of FTO as an RNA m^{6}A demethylase that promotes gene expression through epitranscriptomic regulation.

B) Gene Ontology enrichment analysis identifies multiple pathways activated by FTO overexpression, highlighting the potential of FTO-mediated engineering to enhance T-cell fitness and function and improve the efficacy of T-cell-based immunotherapy.

C) Transcriptomic analysis shows that FTO overexpression significantly increases gene expressions of the DNA helicase CMG complex in engineered T cells, which is responsible for unwinding the DNA double helix and driving DNA replication fork progression.

Supplementary Table S1: List of reagents used in this study.

**List of Reagents**				
**Antibody/Reagents**	**Abbreviation/Name in the text**	**Cat. #**	**Company**	**Species Reactivity**
**MV-4-11**	MV4-11	CRL-9591	ATCC	Human
**Viability Dye eFluor™ 780**	Viability Dye	65086514	eBioscience	
**CD3-PE-Cy7**	CD3	201421	BioLegend	Rat
**CD3-PE**	CD3	201412	BioLegend	Rat
**CD3-PE-Cy7**	CD3	980010	BioLegend	Human
**CD11b/c-FITC**	CD11b/c	201805	BioLegend	Rat

**CD11b/c-APC**	CD11b/c	201809	BioLegend	Rat
**CD11b-APC**	CD11b	982604	BioLegend	Human
**CD90-APC**	CD90	202526	BioLegend	Rat
**CD90-FITC**	CD90	206105	BioLegend	Rat
**CD45-PE**	CD45	202207	BioLegend	Rat
**CD Lineage-FITC (CD3, CD14, CD16, CD19, CD20, CD56)**	CD Lineage	348801	BioLegend	Human
**CD44-PE/Cyanine7**	CD44	338816	BioLegend	Human
**CD34-PERCP**	CD34	340666	BD Biosciences	Human
**CD34-PE**	CD34	340669	BD Biosciences	Human
**CD34-APC**	CD34	128611	BioLegend	mouse
**CD4-APC**	CD4	317416	BioLegend	Human
**CD8-PERCP**	CD8	344708	BioLegend	Human
**CD62L-FITC**	CD62L	324804	BioLegend	Human
**CCR7-PE**	CCR7	353204	BioLegend	Human
**CCR7-APC**	CCR7	353213	BioLegend	Human
**CD15-PE**	CD15	301906	BioLegend	Human
**CD271-PERCP**	PD-L1	46-5983-42	eBioscience	Human
**FTO (fat mass and obesity-associated protein or alpha-ketoglutarate-dependent dioxygenase)**	FTO	45980	Cell Signaling Technology	Human, Mouse, Rat
**RUNX2-Alexa-647**	RUNX2	sc-390351	Santa Cruz Biotechnology	Human, Mouse, Rat
**RANK (CD265)-PE**	RANK	119806	BioLegend	Mouse, Rat
**Osterix (OSX, Sp7)-Alexa-647**	OSX	sc-393325	Santa Cruz Biotechnology	Human, Mouse, Rat
**PE Donkey anti-rabbit IgG**	PE	406421	BioLegend	Rabbit

**PE IgG Isotype**	PE	400111	BioLegend	Mouse
**APC IgG Isotype**	APC	400121	BioLegend	Mouse
**Anti-APC Microbeads**		130090855	Miltenyi Biotec	
**Rat XL Cytokine Array Kit**	Rat Cytokine Array	ARY030	R&D Systems	Rat
**Human XL Cytokine Array Kit**	Human Cytokine Array	ARY022B	R&D Systems	Human
**Seahorse XFp T Cell Metabolic Profiling Kit**		103771-100	Agilent Technologies	
**CCL11 (C-C motif chemokine ligand 11) (Homo sapiens: NM_002986.3)**	CCL11 (EF1a promoter)	EX-Z7461-Lv225	GeneCopoeia	Human
**FTO (fat mass and obesity-associated protein, or alpha-ketoglutarate-dependent dioxygenase) (Rattus norvegicus: NM_001039713.1)**	FTO (CMV promoter)	LPP-Rn18290-Lv105-A00	GeneCopoeia	Rat

Supplementary Table S2: List of Primers (human, OriGene) used in this study.

#	Name	Forward Sequence	Reverse Sequence
1	Rat-β-Actin	TCTTCCAGCCTTCCTTCCTG	CACACAGAGTACTTGCGCTC
2	Rat-CCL11	TTCCATCCCAACTTCCTGCT	TTCTTGGGGTCAGCACAGAT
3	Rat-FTO	TGCGATGATGAAGTGGACCT	CGTATCGGGATCTCTGCCTT
4	Rat-RUNX2	GGGACCGACACAGCCATATA	TCTTAGGGTCTCGGAGGGAA

5	β-Actin (human)	CACCATTGGCAATGAGCGGTTC	AGGTCTTTGCGGATGTCCACGT
6	CCL11 (human)	GCTACAGGAGAATCACCAGTGG	GGAATCCTGCACCCACTTCTTC
7	FTO (human)	CCAGAACCTGAGGAGAGAATGG	CGATGTCTGTGAGGTCAAACGG
8	RUNX2 (human)	CCCAGTATGAGAGTAGGTGTCC	GGGTAAGACTGGTCATAGGACC
9	RANK (human)	GCTCAACAAGGACACAGTGTGC	CGCATCGGATTTCTCTGTCCCA
10	CDK1 (human)	GGAAACCAGGAAGCCTAGCATC	GGATGATTCAGTGCCATTTTGCC
11	CCNA1 (human)	GCACACTCAAGTCAGACCTGCA	ATCACATCTGTGCCAAGACTGGA
12	CCNB1 (human)	GACCTGTGTCAGGCTTTCTCTG	GGTATTTTGGTCTGACTGCTTGC
13	CCNE1 (human)	TGTGTCCTGGATGTTGACTGCC	CTCTATGTCGCACCACTGATACC
